# Epi-microRNA mediated metabolic reprogramming counteracts hypoxia to preserve affinity maturation

**DOI:** 10.1038/s41467-024-54937-0

**Published:** 2024-12-03

**Authors:** Rinako Nakagawa, Miriam Llorian, Sunita Varsani-Brown, Probir Chakravarty, Jeannie M. Camarillo, David Barry, Roger George, Neil P. Blackledge, Graham Duddy, Neil L. Kelleher, Robert J. Klose, Martin Turner, Dinis P. Calado

**Affiliations:** 1 Immunity and Cancer Laboratory, Francis Crick Institute, London, UK; 2 Bioinformatics and Biostatistics Laboratory, Francis Crick Institute, London, UK; 3 Genetic Modification Service Laboratory, Francis Crick Institute, London, UK; 4 Department of Chemistry, Molecular Biosciences and the National Resource for Translational and Developmental Proteomics, Northwestern University, Evanston, IL USA; 5 Advanced Light Microscopy Laboratory, Francis Crick Institute, London, UK; 6 Structural Biology Laboratory, Francis Crick Institute, London, UK; 7 Department of Biochemistry, University of Oxford, Oxford, UK; 8 Immunology Programme, The Babraham Institute, Cambridge, UK

**Keywords:** Germinal centres, B cells, Histone post-translational modifications, Cell growth

## Abstract

To increase antibody affinity against pathogens, positively selected GC-B cells initiate cell division in the light zone (LZ) of germinal centers (GCs). Among these, higher-affinity clones migrate to the dark zone (DZ) and vigorously proliferate by utilizing energy provided by oxidative phosphorylation (OXPHOS). However, it remains unknown how positively selected GC-B cells adapt their metabolism for cell division in the glycolysis-dominant, cell cycle arrest-inducing, hypoxic LZ microenvironment. Here, we show that microRNA (miR)−155 mediates metabolic reprogramming during positive selection to protect high-affinity clones. Mechanistically, miR-155 regulates H3K36me2 levels in hypoxic conditions by directly repressing the histone lysine demethylase, *Kdm2a*, whose expression increases in response to hypoxia. The miR-155-*Kdm2a* interaction is crucial for enhancing OXPHOS through optimizing the expression of vital nuclear mitochondrial genes under hypoxia, thereby preventing excessive production of reactive oxygen species and subsequent apoptosis. Thus, miR-155-mediated epigenetic regulation promotes mitochondrial fitness in high-affinity GC-B cells, ensuring their expansion and consequently affinity maturation.

## Abstract (web summary)

During the antigen-dependent phase of B cell development, clones expressing high-affinity B cell receptors are transferred from light zones to dark zones of germinal centers, while transforming their energy metabolism. Here authors show that in the light zones, the hypoxic microenvironment promotes growth arrest and apoptosis in B cells, while miR-155 protects the high-affinity clones via inducing a switch in energy utilization.

## Introduction

Germinal centers (GC) transiently form in B cell follicles of secondary lymphoid organs upon infection or vaccination. GCs are indispensable sites for increasing the affinity of antibodies, i.e., for the production of high-affinity antibody-secreting plasma cells that are critical for protecting hosts from pathogens. The process by which antibodies increase affinity to the antigen is called affinity maturation^1,2^ and occurs in GCs through a bidirectional migration cycle between two regions, the light zone (LZ) and the dark zone (DZ)^3^. In the LZ, GC-B cells undergo antigen-driven selection in response to signals from B cell receptor (BCR) engagement and T follicular helper cells (TFH)^4^. Positively selected LZ GC-B cells induce *Myc*, a critical cell cycle regulator required for GC maintenance, and transient cMYC expression marks “licensed” GC-B cells^5,6^. Upon receiving positive selection signals, GC-B cells initiate cell division in the LZ. Among these cells, clones with higher-affinity further vigorously proliferate after their transition to the DZ for the clonal expansion^7^. Failure of the positive selection process disrupts GC responses, hinders affinity maturation, and consequently compromises humoral immunity.

Mitochondrial oxidative phosphorylation (OXPHOS) is essential for GC-B cells to proliferate in the DZ^8,9^. In contrast, quiescent GC-B cells are relatively reliant on glycolysis^10^. This is concordant with recent reports showing that the LZ, where predominantly quiescent cells reside, represents a hypoxic microenvironment^11^. Hypoxia promotes glycolysis^12^ and suppresses energy consuming processes, such as cell cycle progression and translation^13–15^. Therefore, cells under hypoxic conditions generally arrest the cell cycle. Nonetheless, engaged discussion surrounds the finding that only LZ GC-B cells reside in a hypoxic microenvironment. This is partly because conventional hypoxia probes are effective only when oxygen levels fall below 1%, and there is a heavy reliance on HIF-1 as a hypoxia marker. These limitations, along with model-specific variations, have led to conflicting reports^8,10,11,16,17^, leaving uncertainty about whether positively selected GC-B cells experience hypoxia.

If the LZ is hypoxic, regardless of the hypoxic status in the DZ, positively selected GC-B cells must undergo metabolic reprogramming from quiescent to proliferative states by counteracting hypoxia. However, such mechanisms remain poorly understood. Moreover, the lack of clarity regarding the hypoxia status in GC-B cells further complicates the understanding of these mechanisms.

Here, we show that microRNA (miR)−155 functions as an epi-miRNA, an epigenetic machinery regulating miRNA, that is essential for metabolic reprogramming in LZ GC-B cells during positive selection. By directly suppressing the hypoxia-induced lysine demethylase 2a (*Kdm2a*), which demethylates H3K36me2 to repress transcription^18,19^, miR-155 promotes mitochondrial remodeling in high-affinity cMyc^+^ GC-B cells under hypoxic conditions. The miR-155- *Kdm2a* interaction enhances OXPHOS by optimizing the expression of key nuclear mitochondrial genes, while preventing excessive production of reactive oxygen species (ROS) and subsequent apoptosis. Hence, miR-155 promotes mitochondrial fitness in high-affinity LZ GC-B cells during positive selection, which allows these cells to endure rapid proliferation upon transitioning to the DZ, thus ensuring affinity maturation.

## Results

### Mitochondrial ROS production is exacerbated by *Mir155*-deficiency in GC-B cells

OXPHOS is required to support the robust proliferation of DZ GC-B cells particularly with high affinity BCRs^9^. Mitochondrial ROS are produced during normal OXPHOS activity at the electron transport chain on the inner mitochondrial membrane^20^. However, defective mitochondria drive overproduction of ROS, which can activate apoptotic pathways^21^. MiR-155 is predominantly expressed in cMyc^+^ GC-B cells and is known to protect cMyc^+^ GC-B cells from apoptosis, particularly those with high-affinity BCRs^22^. Therefore, we hypothesized that miR-155 prevents cell death, in part, by limiting excessive production of mitochondrial ROS.

**Fig. 1:**
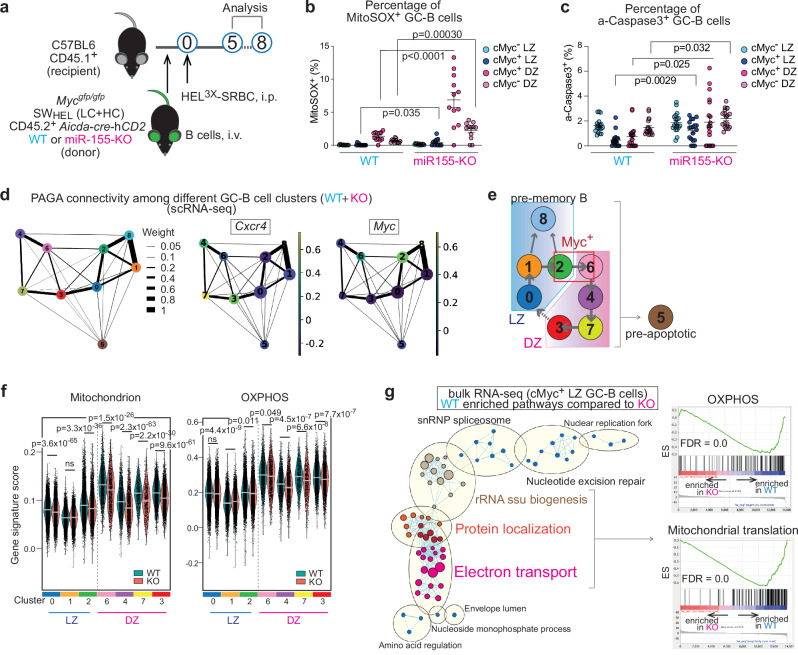
MiR-155 is required for promoting mitochondrial fitness in GC-B cells. **a** Experimental design for all the experiments for Fig. 1. **b** Percentage of MitoSOX^+^ GC-B cells five days after HEL^3 ×^ -SRBC immunization. Pooled from three independent experiments (WT *n* = 12; KO *n* = 12). Unpaired student’s t-test, two-tailed. **c** Percentage of active-Caspase 3^+^ GC-B cells at seven days after HEL^3 ×^ -SRBC immunization. Pooled from two independent experiments (WT *n* = 18; KO *n* = 19). Unpaired student’s t-test, two-tailed. **d** The PAGA algorithm generates a topology-preserving map of single cells along with the sequential changes of gene expression. PAGA graphs were constructed using a k-nearest neighbors’ algorithm to show relationships between clusters. The Ball-and-stick representation displays PAGA connectivity among GC-B cell clusters identified though scRNA-seq. Ball size represents cluster size, and edge thickness indicates connectivity between clusters. Normalized gene expression for *Cxcr4* or *Myc* is shown in the PAGA graph. **e** Predicted relationship between the clusters identified in scRNA-seq of GC-B cells. **f** Violin plots showing gene signature scores for “Mitochondrion” and “OXPHOS”. The bar represents median value. Wilcoxon rank sum test, two-sided, n.s., not significant. **g**, Enrichment map visualizing GSEA results of the bulk RNA-seq data from WT and KO cMyc^+^ LZ GC-B cells. GC-B cells were sorted five days after HEL^3 ×^ -SRBC immunization. WT-enriched pathways (q < 0.0001) were used. Nodes represent gene sets, and edges represent mutual overlap. Highly redundant gene sets were grouped as clusters (left). ES; enriched score, FDR; false discovery rate. Unless otherwise stated, mean ± SEM is indicated.

To test the hypothesis, we utilized the SW_HEL_ adoptive transfer system which allowed us to investigate miR-155 functions in GC-B cells in a B cell-intrinsic manner. In this system, B cells from *Mir155*-sufficient or *Mir155*-deficient SW_HEL_ *Myc*^gfp/gfp^ *Aicda*-cre-hCD2 mice were transferred into CD45.1^+^ congenic recipient mice. SW_HEL_ mice express the hen egg lysozyme (HEL)-specific immunoglobulin heavy chain knock-in and light chain transgene (HyHEL10 BCR)^23^. *Myc*^gfp/gfp^ reporter mice express a GFP-cMYC fusion protein from the endogenous *Myc* locus^24^. *Aicda*-cre-hCD2 transgenic mice carry a Cre gene linked with a truncated human CD2 cDNA via an internal ribosome entry site (IRES) under the control of *Aicda* promoter^25^. In these mice, positively selected cMyc^+^ GC-B cells were identified by GFP fluorescence and hCD2 surface expression. Recipient mice that received transferred B cells were challenged with HEL^3×^ (Fig. 1a). Donor GC-B cells (CD45.1^-^ CD45.2^+^ B220^+^ hCD2^+^ CD38^lo^ CD95^+^) were gated as described in Supplementary Fig. 1a, and by coupling DZ/LZ distinguishing markers (CXCR4/CD86) to cMyc-GFP expression. Then, GC-B cells were subdivided into four consecutive subpopulations: cMyc^-^ LZ, cMyc^+^ LZ, cMyc^-^ DZ and cMyc^+^ DZ^7^. MiR-155 is mostly co-expressed with cMYC in GC-B cells, and the expression of both cMYC and miR-155 changes as follows: highest levels in cMyc^+^ LZ GC-B cells, intermediate levels in cMyc^+^ DZ GC-B cells and followed by negligible levels in cMyc^-^ DZ and cMyc^-^ LZ GC-B cells^5,6,22^ (Supplementary Fig. 1b). Lack of miR-155 did not impact the expression of cMYC in GC-B cells, hence the mean fluorescent intensity (MFI) of cMyc-GFP was comparable between wildtype (hereafter called as “WT”) and *Mir155*-knock out (hereafter called as “KO”) cells (Supplementary Fig. 1c). However, the number of cMyc^+^ LZ and cMyc^+^ DZ GC-B cells were significantly reduced in the absence of miR-155 (Supplementary Fig. 1d). The MitoSOX-based flow cytometric assay for the detection of mitochondrial ROS revealed that in the absence of miR-155, GC-B cell subpopulations, including cMyc^+^ LZ, cMyc^+^ DZ and cMyc^-^ DZ, exhibited a significantly higher percentage of MitoSOX^+^ cells compared to their respective WT cells (Fig. 1b, Supplementary Fig. 1e). Additionally, we observed a concurrent increase in the proportion of active-caspase 3^+^ cells alongside decreased proliferation within the cMyc^+^ LZ, cMyc^+^ DZ and cMyc^-^ DZ subpopulations of the KO mice compared to the WT mice (Fig. 1c, Supplementary Fig. 1f). These findings indicate that miR-155 plays a role in mitigating mitochondrial ROS levels in GC-B cells.

### MiR-155 is required for promoting mitochondrial fitness in GC-B cells

To gain a comprehensive understanding of miR-155-mediated regulation of mitochondrial functions and dynamics of OXPHOS activity during the migration cycle of GC-B cells, we conducted single cell (sc) RNA-seq on both WT and KO GC-B cells and dissected GC-B cells into distinct subpopulations. Unsupervised clustering identified nine distinct clusters: LZ signature genes^9^ were expressed in clusters 0, 1, 2, 5 and 8, and clusters 0 and 1 predominantly represented cMyc^-^ LZ GC-B cells due to their enrichment in the G1 phase of the cell cycle (Supplementary Fig. 1g, h, i). Cluster 8 showed enrichment in pre-memory B cell (pre-MBC)-associated genes, such as *Ccr6*, *Cd38* and *Hhex*^26,27^), indicating its representation of pre-MBC cells (Supplementary Data 1). DZ signature genes^9^ were expressed in clusters 3, 4, 5, 6 and 7 (Supplementary Fig. 1i), which correlated strongly with cell cycle progression, i.e., cluster 6; S phase, cluster 4; S→G2/M phase, cluster 7; G2/M phase and cluster 3; G2/M→G1 phase (Supplementary Fig. 1g). The DZ clusters, 3, 4, 6 and 7 were further analyzed by RNA velocity^28^ to determine a temporal sequence of DZ GC-B cells. This analysis shows the order of the DZ GC-B cell clusters as clusters 6 → 4 → 7 → 3 (Supplementary Fig. 1g).

To establish more specific relationships between the clusters we employed partition-based graph abstraction (PAGA)^29^. The PAGA graph revealed that LZ clusters 0, 1, 2 and 8 formed a closely interconnected group, while DZ clusters 3, 4, 6 and 7, characterized by high expression of Cxcr4, formed a distinct group (Fig. 1d and Supplementary Fig. 1j). Cluster 5, which exhibited higher mitochondrial content, but lower gene counts than the other clusters, did not belong to either LZ or DZ groups (Fig. 1d, Supplementary Fig. 1j, k). Notably, this cluster displayed a significant decrease in rRNA levels (Supplementary Fig. 1l), which is indicative of early apoptosis^30^. Therefore, we excluded cluster 5 from further analysis. The high expression of *Myc* in clusters 2 (LZ) and 6 (DZ) in the PAGA graph corresponded to the enrichment of *Myc* signature genes^5,6^ observed in these clusters, as shown in the dot plot (Fig. 1d and Supplementary Fig. 1i). The increased *Myc* expression in cluster 8 (pre-MBCs) in Fig. 1d is consistent with our previous work suggesting that *Myc*-induced positively selected GC-B cells can give rise to MBCs^7,31^. In the PAGA graph, clusters 0 (LZ) and 3 (DZ) predominantly connect the LZ and DZ groups (Fig. 1d and Supplementary Fig. 1j), indicating that cluster 0 represents LZ B cells soon after migration back from the DZ. The temporal and spatial dynamics of the clusters are summarized in Fig. 1e.

Using these temporally linked GC-B cell clusters, we investigated the changes in mitochondrial functions in GC-B cells in the absence of miR-155 by calculating the gene signature scores for “mitochondrion” and “OXPHOS”. We found these to be significantly lower in cluster 2 (cluster enriched with cMyc^+^ LZ GC-B cells) and all the DZ clusters (clusters 6, 4, 7 and 3) of KO cells than those of WT cells (Fig. 1f). These results suggest that the absence of miR-155 led to impaired mitochondrial functions in cMyc^+^ LZ GC-B cells (cluster 2) and cMyc^+^ DZ GC-B cells (cluster 6), and that such impairment was still observed in cMyc^-^ DZ GC-B cells (clusters 4, 7 and 3) that no longer express miR-155 in WT mice. Thus, miR-155 plays a critical role in maintaining mitochondrial integrity in cMyc^+^ GC-B cells and likely impacts the bioenergetic functions in GC-B cells.

To further clarify the regulatory role of miR-155 in LZ GC-B cells, we carried out bulk RNA-seq analysis on WT and KO cMyc^+^ LZ GC-B cells. Gene set enrichment analysis (GSEA) on RNA-seq data revealed the top eight enriched biological pathways in WT cMyc^+^ LZ GC-B cells compared to KO cMyc^+^ LZ GC-B cells were related to “ribosome”, “translation” and “mitochondria”, either alone or in combination (Supplementary Fig. 1m). Nearly half of the significantly increased genes in the top three pathways (“ribosomal subunit”, “Structural constituent of ribosome” and “ribosome”) encoded mitochondrial ribosomal proteins (Supplementary Data 2). Mitochondrial ribosomal proteins are encoded by nuclear genes and are subsequently transported to the mitochondria following their synthesis in the cytoplasm^32^. The Cytoscape network analysis of GSEA results revealed an enrichment of pathways involved in mitochondrial protein transport and mitochondrial functions, particularly electron transport, in WT cMyc^+^ LZ GC-B cells compared to their KO counterparts (Fig. 1g). This finding indicates that these pathways are compromised or defective in KO cMyc^+^ LZ GC-B cells. In line with the enrichment map, genes associated with “OXPHOS”, and “Mitochondrial translation” exhibited a significant enrichment in WT cMyc^+^ LZ GC-B cells compared to KO cMyc^+^ LZ GC-B cells, suggesting impaired functionality of these pathways in KO cMyc^+^ LZ GC-B cells (Fig. 1g). To assess metabolism in GC-B cells, we conducted SCENITH, which allows metabolic profiling by flow cytometry^33^. The results showed that KO cMyc^+^ GC-B cells exhibited increased glucose dependence (i.e., reliance on glycolysis and OXPHOS) and reduced fatty acid and amino acid oxidation (FAO/AAO) compared to their WT counterparts (Supplementary Fig. 1n). This metabolic profile is consistent with that observed in GC-B cells from transcription factor A, mitochondrial (*Tfam*)-deficient mice, which have defective mitochondria^34^. These findings strongly indicate impaired mitochondria in KO cMyc^+^ GC-B cells. Collectively, our findings suggest that mitochondrial fitness is exclusively established in the LZ during positive selection and that miR-155 expressed in the LZ is critical for maintaining mitochondrial bioenergetic functions in GC-B cells.

### MiR-155 regulates H3K36me2 levels in dividing LZ GC-B cells

**Fig. 2:**
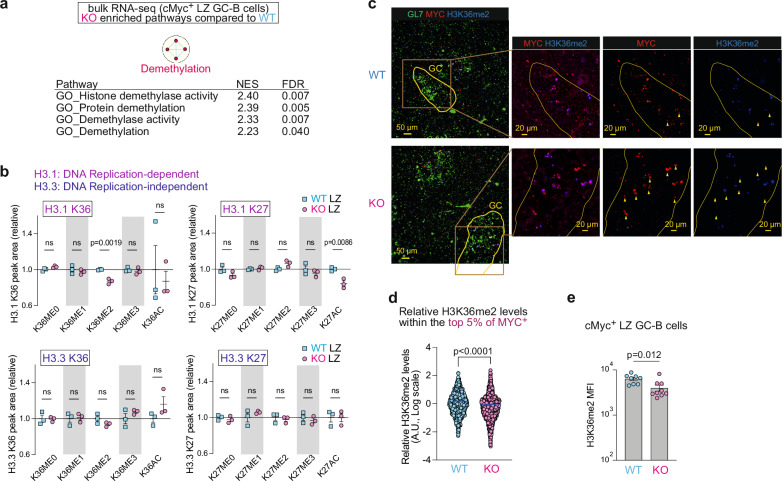
MiR-155 regulates H3K36me2 levels in dividing LZ GC-B cells. **a** Enrichment map visualizing GSEA results of bulk RNA-seq data from WT and KO cMyc^+^ LZ GC-B cells. KO-enriched pathways (q < 0.1) were used. **b** The relative abundance of histone PTMs, including histone H3.1 K36, H3.3 K36, H3.1 K27 and H3.3 K27 in LZ GC-B cells from WT and KO mice. Donor-derived GC-B cells were sorted five days after HEL^3 ×^ -SRBC immunization for analysis. Triplicate samples were used for both WT and KO backgrounds. Unpaired student’s t-test, two-tailed. **c** Splenic sections of C57BL/6 mice, in the presence and absence of miR-155, seven days after SRBC immunization were stained for GL7 (green), MYC (red) and H3K36me2 (blue). Yellow wedges represent cells that are positive for MYC but negative for KDM2A. Scale bar, 50 µm. Representative images from two experiments (WT *n* = 4; KO *n* = 4). **d** Relative H3K36me2 levels determined by calculating the ratio of H3K36me2 MI to MYC MI from Supplementary Fig. 2c. The top 5% of MYC intensity values, representing the highest MYC expressing cells, were selected from each batch of two independent experiments. The bar represents mean. Unpaired student’s t-test, two-tailed. A.U., arbitrary unit. **e** MFI of H3K36me2 in cMyc^+^ LZ GC-B cells. Pooled from three independent experiments (WT *n* = 8; KO *n* = 9). Unpaired student’s t-test, two-tailed. Unless otherwise stated, mean ± SEM is indicated. n.s., not significant.

To visualize the enriched pathways in KO cells, we generated a Cytoscape enrichment map using RNA-seq data from cMyc^+^ LZ GC-B cells. The enrichment map revealed that the gene signature “Demethylation” was the only pathway significantly enriched in KO cMyc^+^ LZ GC-B cells when compared to WT cells (Fig. 2a). The signature “Demethylation” represents the process of removing one or more methyl groups from a molecule and consisted of four pathways in this comparison: “Histone demethylase activity”, “Protein demethylation”, “Demethylase activity”, and “Demethylation” (Fig. 2a, Supplementary Data 3). Therefore, we investigated whether there were any alterations in histone post-translational modifications (PTM) in KO GC-B cells compared to WT GC-B cells. To this end, we employed a liquid chromatography with tandem mass spectrometry (LC-MS/MS) technique that allows unbiased identification and quantitative profiling of histone PTMs^35,36^. This method successfully quantified the abundance of histone PTMs in both WT and KO LZ GC-B cells, including methylation and acetylation on histone H1.4, H3 and H4. The analysis showed a significant decrease in di-methylation of histone H3.1 at lysine 36 (K36me2) and an increase in unmethylated histone H3 at lysine 23 (K23me0) in KO LZ GC-B cells compared to WT LZ GC-B cells (Fig. 2b and Supplementary Fig. 2a). We also observed a decrease in acetylation of H3 at K23 (K23ac) (Supplementary Fig. 2a), which is linked to transcriptional activation^37^. Acetylation of H3 at K23 can be catalyzed by cyclic adenosine monophosphate response element-binding protein binding protein (CREBBP), monocytic leukemia zinc finger protein (MOZ) complex and its paralog monocytic leukemia zinc finger related factor (MORF) complex^37,38^. However, there were no differentially expressed genes (DEG) in the bulk RNA-seq dataset between KO and WT cMyc^+^ LZ-GC-B cells for any genes encoding protein products that are part of these complexes (Supplementary Data 2). Therefore, it was unlikely that the absence of miR-155 was directly responsible for the decreased H3K23ac levels in KO LZ GC-B cells. Because the reduction of H3K23ac could lead to increased levels of unmethylated H3K23, the elevation in H3K23me0 levels appears to be not directly caused by the lack of miR-155 either. Thus, the decreased levels of K36me2 on histone H3.1 appeared to be the sole altered histone methylation directly impacted by the absence of miR-155-mediated regulation in KO cMyc^+^ LZ GC-B cells.

The reduction of K36me2 levels was specific to DNA replication-dependent histone H3.1 and was not observed in DNA replication-independent histone H3.3 (Fig. 2b). This suggests that cell division is required for the depletion of H3K36me2. Previous studies have indicated that the loss of H3K36me2 is associated with a decrease in acetylation of histone H3 at K27 (K27ac)^39^. There was decreased K27ac on histone H3.1 in KO LZ GC-B cells relative to WT cells, while no such change was observed on histone H3.3 (Fig. 2b). The decrease in K36me2 on histone H3.1 may lead to increased levels of monomethylated K36 (K36me1) on histone H3.1. While no elevation of K36me1 was detected on histone H3.1 in KO LZ GC-B cells relative to their WT cells, a significant increase in this histone PTM was observed in KO DZ GC-B cells compared to their WT counterparts (Fig. 2b and Supplementary Fig. 2b). This could be attributed to the migration of dividing LZ GC-B cells to the DZ, where chromatin marks could potentially be deposited on newly synthesized histones. Apart from K36me1, no other changes were observed in the histone PTMs at K36 of histone H3.1 in DZ GC-B cells between WT and KO cells (Supplementary Fig. 2b). These findings suggest that miR-155 regulates the levels of K36me2 of H3.1 during cell division in LZ GC-B cells. Given that LZ GC-B cells are generally quiescent, these dividing cells likely represent cMyc^+^ LZ GC-B cells^7^.

To examine the effect of miR-155-mediated regulation on histone H3K36me2 levels in cMyc^+^ GC-B cells, we performed immunostaining of spleen sections from SRBC-immunized, *Mir155*-sufficient and *Mir155*-deficient mice. Within the GL7-positive GC areas, a correlation in co-expression of MYC and H3K36me2 was observed in both groups to some extents (Fig. 2c, Supplementary Fig. 2c). However, the Pearson correlation coefficients with 95% confidence interval showed no overlap between the values of WT and KO groups (r = 0.574 – 0.596 and 0.344 – 0.384, for WT and KO, respectively), indicating a stronger correlation in the WT group than the KO group (Supplementary Fig. 2c). To investigate the difference in H3K36me2 levels between WT and KO MYC^+^ GC-B cells, we compared the relative mean intensity of H3K36me2 in cells within the top 5% of MYC positive cells from the total cell population shown in Supplementary Fig. 2c. The analysis revealed a significant reduction in H3K36me2 levels in KO MYC^+^ GC-B cells compared to the corresponding WT cells (Fig. 2d). This reduction of H3K36me2 levels in KO cMyc^+^ LZ GC-B cells was also confirmed by flow cytometry (Fig. 2e). Consistent with the results derived from the MS analysis (Fig. 2b), total LZ GC-B cells in KO background showed reduced H3K36me2 levels compared to WT cohorts, whereas total DZ GC-B cells did not show this reduction (Supplementary Fig. 2d). We also performed immunofluorescence-based correlation analysis between MYC and H3K36me1 or H3K36me3. However, the correlation in co-expression of MYC and these histone PTMs exhibited only minor variations when comparing KO and WT MYC^+^ GC-B cells (Supplementary Fig. 2e, f). These results suggest that the co-expression pattern between MYC and H3K36me1 or H3K36me3 remains largely unchanged, unlike the substantial changes observed in H3K36me2. Collectively, these results provide compelling evidence for the crucial role of miR-155 as an epigenetic modulator in histone PTMs. Notably, miR-155 predominantly regulates the levels of H3K36me2 in dividing cMyc^+^ LZ GC-B cells, potentially this may influence mitochondrial functions in GC-B cells.

### MiR-155 primarily controls *Kdm2a* expression in high-affinity cMyc^+^ LZ GC-B cells

**Fig. 3:**
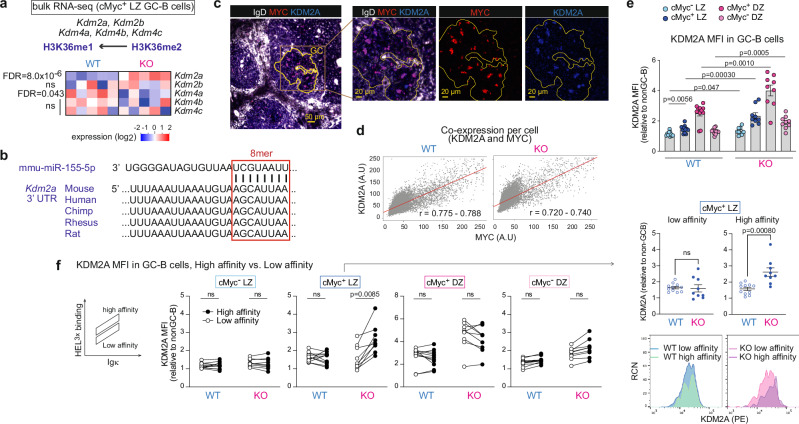
MiR-155 primarily controls KDM2A expression in high affinity cMyc^+^ LZ GC-B cells. **a** Heat map showing the transcript levels of the enzymes involved in the demethylation of histone H3K36me2. The bulk RNA-seq dataset from WT and KO cMyc^+^ LZ GC-B cells was used. FDR values are shown. **b** Sequence alignment of a part of the 3’UTR of *Kdm2a*. The 8-mer miR-155 binding site is highlighted in a box. **c** Immunofluorescence staining of splenic section from a WT mouse seven days after SRBC immunization, showing IgD (white), MYC (red) and KDM2A (blue). Scale bar, 50 µm. Representative section images from two experiments (WT *n* = 4; KO *n* = 4). **d** Scatter plots show the MI of MYC and KDM2A within segmented GC cells in spleen sections from immunized WT and KO mice. The Pearson correlation coefficient (r) is presented with a 95% confidence interval in red. A.U., arbitrary unit. **e** Relative MFI of KDM2A in cells from four GC-B cell subpopulations. GC-B cells were derived from WT or KO donor B cells seven days after HEL^3 ×^ -SRBC immunization. Pooled from two independent experiments (WT *n* = 11; KO *n* = 9). Unpaired student’s t-test, two-tailed. **f** Relative MFI of KDM2A in cells from four GC-B cell subpopulations. GC-B cells were derived from WT or KO donor B cells seven days after HEL^3 ×^ -SRBC immunization. Low and high affinity were determined based on HEL^3 ×^ binding within Igκ^+^ GC-B cells. Pooled from two independent experiments (WT *n* = 11; KO *n* = 9). RCN, relative cell number. Paired student’s t-test, two-tailed. Unless otherwise stated, mean ± SEM is indicated. n.s., not significant.

Demethylation of H3K36me2 can be catalyzed by the lysine demethylases (KDMs), *Kdm2a, Kdm2b, Kdm4a, Kdm4b and Kdm4c*^40^. We examined whether any of mRNAs encoding these enzymes was differentially expressed between WT and KO cMyc^+^ LZ GC-B cells. Expression of *Kdm2a* was significantly higher in KO cMyc^+^ LZ GC-B cells compared to WT cMyc^+^ LZ GC-B cells, while the others (*Kdm2b, Kdm4b* and *Kdm4c*) exhibited similar expression levels between the two groups (Fig. 3a). In contrast, the expression of *Kdm4a* was found to be decreased in KO cMyc^+^ LZ GC-B cells compared to WT cMyc^+^ LZ GC-B cells (Fig. 3a and Supplementary Data 2). Next, we examined whether *Kdm2a* mRNA is predicted as a target of miR-155. By utilizing miR prediction tools, we identified *Kdm2a* as one of the 18 genes that were predicted to be targets of miR-155 and showed differential expression between WT and KO cMyc^+^ LZ GC-B cells (Supplementary Fig. 3a and Supplementary Data 2). *Kdm2a* has one 8-mer miR-155-binding site at 3’ UTR that is conserved across multiple species, including human, mouse, and rat (Fig. 3b).

To investigate the relationship between KDM2A and miR-155, we examined the co-expression of KDM2A and MYC by immunostaining tissue sections derived from *Mir155*-sufficient and *Mir155*-deficient mice using antibodies against IgD, MYC and KDM2A. This revealed a strong correlation in co-expression of these two proteins in both groups (r = 0.775–0.788 and 0.720–0.740, for WT and KO, respectively) (Fig. 3c, d). Therefore, we concluded that KDM2A is mainly expressed in positively selected cMyc^+^ GC-B cells. To examine the degree and cell subpopulations expressing KDM2A, we performed flow cytometric analysis of GC-B cells derived from SW_HEL_ adoptive transfer system. In WT mice, the analysis revealed a ~ 1.3-fold increase in KDM2A expression in cMyc^+^ LZ GC-B cells compared to cMyc^-^ LZ GC-B cells (Fig. 3e), indicating that positive selection signals can induce its expression. The highest expression of KDM2A was observed in cMyc^+^ DZ GC-B cells. A similar expression pattern was observed in KO GC-B cell subpopulations. In all GC-B cell subpopulations, KDM2A expression was significantly higher in KO cells compared to their WT counterparts (Fig. 3e and Supplementary Fig. 3b).

The SW_HEL_ system enables the distinction between high and low affinity B cells following HEL^3×^ immunization. When comparing high and low affinity clones within each WT GC-B cell subpopulation, there were no significant differences in the level of KDM2A (Fig. 3f). In contrast, the lack of miR-155 led to a substantial increase of KDM2A expression in high affinity clones compared to low affinity clones within the cMyc^+^ LZ GC-B cell subpopulation. As a result, there was a marked elevation in KDM2A expression in high affinity clones of KO cells compared to WT cells (Fig. 3f). Collectively, these results suggest that KDM2A is preferentially induced in high affinity clones within the cMyc^+^ LZ GC-B cell subpopulation during positive selection, and therefore, miR-155 is required to dampen KDM2A expression in these cells.

### Hypoxia induces KDM2A in LZ GC-B cells

**Fig. 4:**
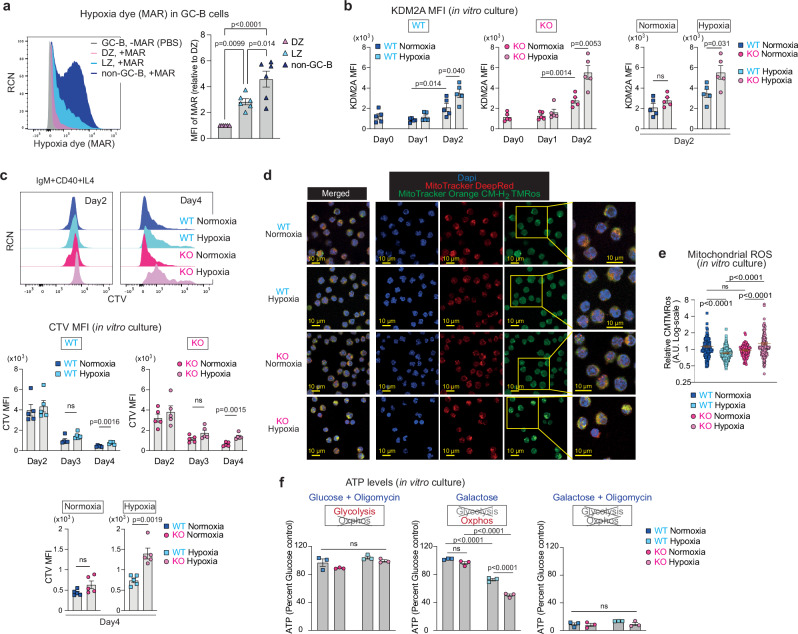
The regulatory activity of miR-155 is revealed under hypoxia. **a** Representative flow cytometric histograms of hypoxia dye, MAR vs. relative cell number (RCN) (left). Relative MFI of MAR (right) in GC-B and non-GC-B cells. GC-B and non-GC-B cells were isolated from inguinal lymph nodes of C57BL/6 mice. One-way ANOVA. Pooled from three independent experiments (*n* = 6). **b** KDM2A MFI of B cells from WT and KO mice cultured under normoxia (21% O_2_) or hypoxia (1% O_2_) for the indicated time periods. Unpaired student’s t-test, two-tailed. Pooled from three independent experiments (WT *n* = 5; KO *n* = 5). **c** Representative flow cytometric histograms of CTV vs. RCN at day 2 and 4 (top). CTV MFI of B cells from WT and KO mice cultured under normoxia or hypoxia for the indicated time periods (bottom). Unpaired student’s t-test, two-tailed. Pooled from five independent experiments (WT *n* = 5; KO *n* = 5). **d** Representative image of B cells from WT and KO mice cultured under normoxia or hypoxia for two days, followed by staining with MitoTracker Deep Red (Red) and MitoTracker Orange CM-H_2_ TMRos (green). DAPI (blue) was used as a nuclear counterstaining. Scale bar, 10 µm. Representative images are shown. **e** Quantification of relative MitoTracker Orange CMTMRos levels. Each dot represents one cell. One-way ANOVA. Pooled from two independent experiments (WT *n* = 4; KO *n* = 4). **f** Relative ATP levels in B cells from WT and KO mice. The replated cells were cultured for two hours in hypoxia with indicated media for ATP measurement. The luminescence values of each type of B cells cultured in media containing glucose for two hours in hypoxia were used to calculate relative ATP levels. One-way ANOVA. Pooled data from three experiments (WT *n* = 3; KO *n* = 3). Unless otherwise stated, mean ± SEM is indicated. n.s., not significant.

Hypoxia induces *Kdm2a* expression at both transcript and protein levels^41,42^. Given that previous studies have shown that the LZ of GCs is hypoxic^11^, we hypothesized that miR-155 counteracts the hypoxia-induced increase of KDM2A expression: i.e., in the absence of miR-155, KDM2A expression is enhanced under hypoxic conditions. Since the concept that the LZ has a more hypoxic microenvironment than the DZ remains a subject of controversy^8,16^, we quantified the degree of hypoxia that GC-B cells were experiencing using the hypoxia detecting dye, MAR (see “methods”), which effectively differentiated between hypoxic and normoxic B cells (Supplementary Fig. 4a). Flow cytometric analysis revealed that a bimodal distribution of MAR fluorescence intensity in non-GC-B cells. This potentially reflects their location within the tissue, as regions adjacent to blood vessels are generally considered less hypoxic, whereas areas distant from blood vessels experience more hypoxia^43^. Additionally, differences in detection sensitivity between MAR (which detects hypoxia at ~5% O_2_)^44^ and conventional hypoxia probes, such as pimonidazole (which detects hypoxia at <1% O_2_)^45^, may contribute to the detection of non-GC-B cells as hypoxic. Here, LZ GC-B cells exhibited higher MAR fluorescence intensity than DZ GC-B cells (Fig. 4a), indicating greater levels of hypoxia, in line with previous studies^10,11,17^.

To examine the effect of hypoxia on KDM2A expression in B cells, we cultured splenic B cells in the presence of anti-IgM, anti-CD40 and IL-4, which mimicked the combination of positive selection signals derived from BCR and T cell help. These cells were cultured in either normal oxygen concentration (21%, normoxic) or low oxygen concentration (1%, hypoxic), and KDM2A expression was examined by flow cytometry. Following a two-day activation with positive selection mimics under normoxic conditions, both WT and KO B cells showed increased KDM2A expression (Fig. 4b). However, there was no significant difference in KDM2A levels between WT and KO B cells under normoxic conditions (Fig. 4b). Under hypoxic conditions, the expression of KDM2A was markedly increased, resulting in higher expression of KDM2A in both WT and KO B cells compared to normoxic conditions (Fig. 4b). By contrast, in hypoxic cultures, KDM2A expression was ~1.6-fold greater in the KO B cells than WT B cells (Fig. 4b). This difference in expression was attributed to the increased expression of miR-155 under hypoxic conditions (Supplementary Fig. 4b), consistent with previous reports^46,47^. Therefore, hypoxia was necessary for observing differential levels of KDM2A between KO and WT in vitro cultured B cells. These findings suggest that miR-155 exerts a strong repressive effect on *Kdm2a* primarily under hypoxic conditions.

### The regulatory activity of miR-155 is revealed under hypoxia

To examine the impact of hypoxia on cell division, we cultured CellTraceViolet (CTV)-labelled B cells from *Mir155*-sufficient and *Mir155*-deficient mice with positive selection mimics in either normoxic or hypoxic conditions. As expected, hypoxia suppressed cell proliferation regardless of genotype (Fig. 4c). However, whereas both WT and KO B cells showed similar levels of division in normoxic cultures, miR-155 KO B cells exhibited significantly less division than WT B cells under hypoxic conditions (Fig. 4c). In contrast to ex vivo GC-B cells, the levels of apoptosis in WT cells and KO B cells were similar in vitro (Supplementary Fig. 4c, Fig. 1c). The results highlight the critical role of miR-155 for cell proliferation under hypoxic conditions.

To investigate the influence of hypoxia on mitochondrial ROS production, we used non-fluorescent MitoTracker Orange CM-H_2_ TMRos that becomes fluorescent upon oxidization. After a two-day culture period under either hypoxic or normoxic conditions, we stained WT and KO B cells with MitoTracker dyes. WT B cells produced lower amounts of mitochondrial ROS under hypoxic conditions (Fig. 4d, e). In contrast, hypoxic KO cultures had significantly higher amounts of mitochondrial ROS than normoxic KO cultures (Fig. 4d, e), indicating mitochondrial dysfunction in these cells. When we compared the size of WT and KO B cells cultured under normoxia and hypoxia by flow cytometry, both WT and KO cells showed equally reduced cell size under hypoxia compared to normoxia (Supplementary Fig. 4d). Despite the reduced size of hypoxic cultures compared to normoxic cultures, we also observed increased ROS in hypoxic KO cultures compared to normoxic KO cultures through a MitoSOX-based flow cytometric assay (Supplementary Fig. 4e). Consequently, KO B cells exhibited a significantly increased ROS production compared to WT B cells under hypoxic conditions, while no such difference was observed under normoxic conditions (Fig. 4d, e, Supplementary Fig. 4e). To further investigate mitochondrial integrity in KO B cells, we assessed energy metabolic states coupled to mitochondrial functions. To this end, we measured the abundance of adenosine triphosphate (ATP) produced through metabolic processes. After two days of culturing WT and KO B cells under either normoxic or hypoxic conditions, we further cultured the cells in the following media supplemented with either glucose, glucose plus oligomycin, galactose, or galactose plus oligomycin. To directly compare energy metabolic capacities of cells, all cells were cultured in hypoxic conditions for 2 h using the indicated media. Cells can use both glycolysis and OXPHOS to produce ATP when cultured in glucose-supplemented medium, while in medium supplemented with glucose and oligomycin, an inhibitor of OXPHOS, cells rely solely on glycolysis for ATP production. Under hypoxic conditions, both WT and KO cells exhibited a reduction of ATP abundance when cultured in glucose-supplemented medium, compared to their normoxic counterparts (Supplementary Fig. 4f). This result is consistent with previous reports showing reduced energy production under hypoxia^48,49^. When B cells were cultured in medium supplemented with both glucose and oligomycin, the relative abundance of ATP was similar to corresponding cultures supplemented with glucose alone across all cell types (Fig. 4f), indicating that the cells were able to survive by relying on their glycolytic capacity without the need for OXPHOS, in accordance with the well-known “Crabtree effect”^50,51^. In contrast, cells cultured in galactose-supplemented medium must use OXPHOS to generate ATP^52^. In this culture condition, we observed a reduction in ATP abundance in both WT and KO B cells under hypoxia compared to normoxia.

Furthermore, while ATP production through OXPHOS was compromised in hypoxic cultures of both WT and KO cells, the impairment was more severe in KO B cells (Fig. 4f). ATP production was abrogated when WT or KO B cells were cultured in medium containing galactose and oligomycin regardless of oxygen tension (Fig. 4f). These results suggest that miR-155 plays a critical role in the metabolic reprogramming of activated B cells by regulating OXPHOS under hypoxic conditions. Collectively, the results demonstrate that the regulatory activity of miR-155 in modulating mitochondrial function and energy metabolism in activated B cells is manifested in a hypoxic environment.

### MiR-155 optimizes nuclear mitochondrial gene expression through KDM2A-mediated control of H3K36me2 levels

**Fig. 5:**
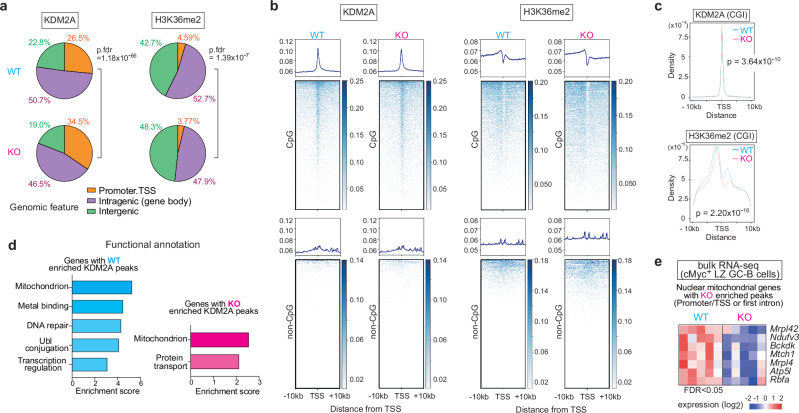
MiR-155 optimizes nuclear mitochondrial gene expression through *Kdm2a*-mediated control of H3K36me2 levels. **a** Genomic distribution of KDM2A and H3K36me2 ChIP-seq peaks in B cells from WT and KO mice cultured for two days under hypoxia is shown as pie charts. The average percentage of the ChIP-seq triplicate samples is displayed. Promoter regions are defined as ± 2 kb of the transcription start site (TSS). Two-sided, p.FDR values are shown. **b** Metagene analysis and heatmaps of KDM2A or H3K36me2 for CpG islands (CGIs) containing genes or genes without CGIs. Metagene analyses are displayed in ± *10* *kb* windows. A representative sample is shown. **c** Plots showing the distribution of peak distance to the nearest TSS. The average ChIP-seq read occupancy for KDM2A or H3K36me2 surrounding the TSS of CGIs containing genes. Kolmogorov-Smirnov test. *p*-values are shown. **d** Analysis of genes with “WT-enriched” and “KO-enriched” KDM2A peaks using the DAVID Functional Annotation Clustering Tool. Pathways with a two-sided modified fisher exact *p*-value < 0.05 were used for the analysis. **e** A heat map showing transcript levels of seven mitochondrial genes with “KO-enriched” KDM2A peaks at the promoter/TSS regions or first introns near the TSS.

To investigate how *Mir155*-deficiency impacts genome-wide binding of KDM2A and H3K36me2 distribution, we performed ChIP-seq assays using hypoxic B cell cultures. The genomic distribution of KDM2A and H3K36me2 peaks showed significant differences between WT and KO B cells (Fig. 5a). In KO B cells compared to WT B cells, there was a proportional increase in KDM2A peaks within the promoter/transcriptional start site (TSS) regions (WT = 26.5%, KO = 34.5%), accompanied by a concurrent reduction of H3K36me2 peaks in the same regions (WT = 4.59%, KO = 3.77%) (Fig. 5a). Consistent with a previous report showing that KDM2A preferentially binds to CGIs islands (CGIs) primarily located at gene promoters^53^, we detected significant enrichment of KDM2A peaks specifically at the TSS-proximal regions of genes containing CGIs (Fig. 5b). In agreement with these results, when the levels of H3K36me2 within 2.5 kb downstream of a TSS of genes containing CGIs were calculated and presented as a ratio of average KO to WT for each gene, KO B cells exhibited lower H3K36me2 levels than WT B cells in the TSS regions of 17,618 genes. In contrast, KO B cells exhibited higher H3K36me2 levels in the TSS regions of only 6,724 genes (Supplementary Data 4). Furthermore, we observed a concurrent depletion in H3K36me2 signals within these regions of CGI containing genes in both WT and KO B cells (Fig. 5b). To identify distinctive patterns of KDM2A binding and H3K36me2 marks, we examined the peak positions in relation to the nearest TSS (Fig. 5c). These plots revealed that a significantly higher density of KDM2A peaks in KO B cells at TSS-proximal regions compared to WT B cells (Fig. 5c). Accordingly, H3K36me2 peak density was significantly reduced in KO B cells relative to WT B cells at these same regions and in the intragenic regions proximal to the TSS (Fig. 5c). Our findings indicate that the absence of miR-155 leads to an elevated expression of KDM2A, which in turn triggers enhanced binding of KDM2A to TSS-proximal regions and a concomitant depletion of H3K36me2 in those regions as well as in the intragenic regions.

To elucidate the biological functions of genes bound by KDM2A, we investigated the KDM2A target genes that displayed differential binding between WT and KO B cells and identified “WT-enriched” and “KO-enriched” KDM2A peaks (Supplementary Data 5). Functional annotation clustering analysis with DAVID^54^ showed that genes with “WT-enriched” or “KO-enriched” KDM2A peaks were strongly associated with the regulation of mitochondrial functions (Fig. 5d). These results suggest that KDM2A plays a role in regulating the expression of nuclear mitochondrial genes during hypoxia. Among the genes with “WT-enriched” and “KO-enriched” KDM2A peaks, we identified 102 and 30 genes encoding nuclear mitochondrial proteins listed in MitoCarta 3.0^55^, respectively. Bulk RNA-seq analysis showed that five mitochondrial genes with “KO-enriched” KDM2A peaks at promoter/TSS regions and two mitochondrial ribosomal proteins with “KO-enriched” KDM2A peaks at the first intron, which corresponds to the intragenic regions proximal to the TSS, were reduced in their expression in KO cMyc^+^ LZ GC-B cells compared to WT cMyc^+^ LZ GC-B cells (Fig. 5e and Supplementary Data 2). The nuclear mitochondrial genes with “KO-enriched” KDM2A peaks exhibited a concomitant decrease in H3K36me2 levels (Supplementary Fig. 5a). These findings suggested that the differential binding of KDM2A between WT and KO B cells affected the expression of nuclear mitochondrial genes. Furthermore, among the five genes with “KO-enriched” KDM2A peaks located in the promoter/TSS regions, *Ndufv3* and *Atp5l* are known to play an essential role in OXPHOS^55^. Thus, the miR-155-*Kdm2a* axis has a critical function in regulating OXPHOS coupled with mitochondrial bioenergetic functions in activated B cells under hypoxic conditions.

### The miR-155-*Kdm2a* interaction regulates mitochondrial ROS and apoptosis in GC-B cells

**Fig. 6:**
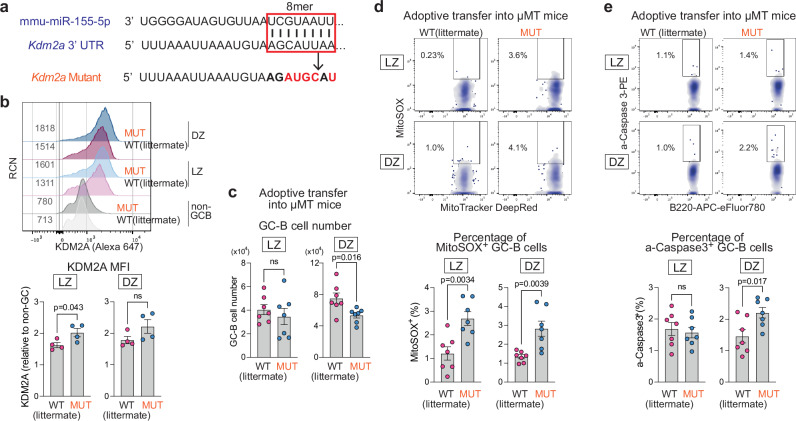
The miR-155-*Kdm2a* interaction regulates mitochondrial ROS and apoptosis in GC-B cells. **a** Schematics representation of the 8-mer miR-155-binding site in the 3’UTR of the *Kdm2a* (boxed) and the introduced base changes (red letters) to disrupt miR-155 binding in *Kdm2a* mutant mice. **b** Representative flow cytometric histograms showing KDM2A vs. RCN (top). RCN, relative cell number. Relative MFI values of KDM2A in LZ and DZ GC-B cells are shown (bottom). GC-B cells were derived from WT and MUT mice seven days after SRBC immunization. Unpaired student’s t-test, two-tailed. Pooled from three independent experiments (WT *n* = 4; MUT *n* = 4). **c** Number of LZ and DZ GC-B cells from WT and MUT mice within the µMT adoptive transfer system. Unpaired student’s t-test, two-tailed. Pooled from two independent experiments (WT *n* = 7; MUT *n* = 7). **d** Representative flow cytometric plots of MitoTracker DeepRed vs. MitoSOX in GC-B cells from WT and MUT mice within the µMT adoptive transfer system (top). Percentage of MitoSOX^+^ GC-B cells (bottom). Unpaired student’s t-test, two-tailed. Pooled from two independent experiments (WT *n* = 7; MUT *n* = 7). **e** Representative flow cytometric plots of B220 vs. active-Caspase 3 in GC-B cells from WT and MUT mice within the µMT adoptive transfer system (top). Percentage of active-Caspase 3^+^ GC-B cells (bottom). Unpaired student’s t-test, two-tailed. Pooled from two independent experiments (WT *n* = 7; MUT *n* = 7). Unless otherwise stated, mean ± SEM is indicated. n.s., not significant.

To validate the regulatory interaction of the miR-155-*Kdm2a* axis, we generated mice with a mutated miR-155 binding site in the 3’UTR of *Kdm2a* (Fig. 6a). B cell development was normal in homozygous mutant (hereafter called “MUT”) mice (Supplementary Fig. 6a). WT and MUT mice were immunized with SRBCs, and KDM2A expression in GC-B cells was assessed by flow cytometry. In LZ GC-B cells of MUT mice, KDM2A expression exhibited a ~ 1.2-fold increase relative to their WT littermates (Fig. 6b). No significant difference in KDM2A expression was observed in DZ GC-B cells. These results indicate that miR-155 limits *Kdm2a* expression in LZ GC-B cells through its binding site on *Kdm2a* mRNA. To examine B cell-intrinsic effects of the mutation, we performed adoptive transfer experiments by transferring B cells isolated from the spleens of either WT or MUT mice into µMT mice, which lacks mature B cells^56^. The mice were then immunized with SRBCs. Seven days later, all recipient mice gave rise to GC-B cells, but the total number of DZ GC-B cells was reduced by ~29% in the MUT background compared to that of WT, while the number of LZ GC-B cells was similar between WT and MUT mice (Fig. 6c and Supplementary Fig. 6b, c).

We investigated the impact of the 3’UTR mutation on mitochondrial ROS and apoptosis in GC-B cells by transferring B cells into µMT mice. The proportion of MitoSOX^+^ cells was higher in both LZ and DZ GC-B cells of MUT mice compared to WT (Fig. 6d). Moreover, the percentage of active-Caspase3^+^ cells were increased in DZ GC-B cells of MUT mice, when compared to WT mice (Fig. 6e). The elevated apoptosis in GC-B cells from MUT mice compared to WT mice provides an explanation for the decreased number of DZ GC-B cells depicted in Fig. 6c. These results largely mirror the observations in *Mir155*-deficient mice. Moreover, the overexpression of KDM2A in GC-B cells through lentiviral transduction resulted in an increased percentage of MitoSOX^+^ DZ GC-B cells compared to control cells (Supplementary Fig. 6d). Taken together, these findings underscore the crucial role of miR-155 in repressing *Kdm2a* to counteract the effects of hypoxia during the positive selection process.

## Discussion

**Fig. 7:**
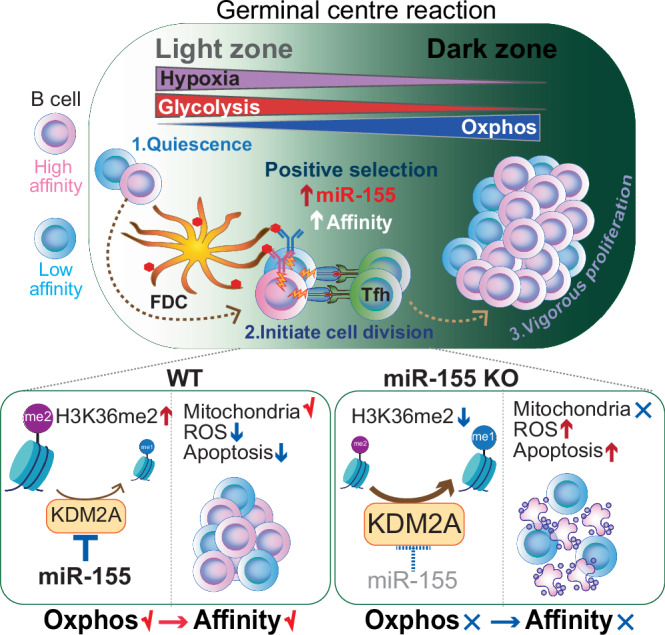
Working model: miR-155-mediated epigenetic regulation control mitochondrial fitness during GC-B cell positive selection. Following the reception of signals through BCR engagement and T cell help, LZ GC-B cells undergo a positive selection process that induces cell division. Positively selected GC-B cells, particularly high-affinity clones, migrate to the less hypoxic DZ and sustain their vigorous cell division by relying on OXPHOS for energy production. To promote the dynamic transition of GC-B cells from the hypoxic LZ to the DZ under hypoxia-induced stress, miR-155 plays a crucial role by repressing the hypoxia-induced expression of KDM2A. This repression mechanism enables precise regulation of H3K36me2 levels, leading to optimal expression of nuclear mitochondrial genes. As a result, mitochondrial remodeling is facilitated, and OXPHOS is enhanced in hypoxic conditions. Thus, the miR-155-*Kdm2a* interaction is indispensable for promoting mitochondrial fitness during positively selection, which provides robust mitochondrial in high-affinity clones to endure the high-rate of cell division in the DZ. This mechanism allows high-affinity clones to undergo clonal expansion, thus ensuring the process of affinity maturation.

We demonstrated that miR-155 critically regulates H3K36me2 levels by directly repressing histone demethylase *Kdm2a* during GC positive selection. This repression enabled GC-B cells to reprogram their metabolism through the remodeling of mitochondria and to initiate cell division in the hypoxic LZ microenvironment. Maintaining mitochondrial fitness during positive selection in the LZ was essential for higher-affinity GC-B cells to undergo clonal expansion in the DZ, consequently ensuring affinity-maturation in GC responses. In the absence of miR-155, dysfunctional mitochondria resulted in increased ROS production and subsequent apoptosis in GC-B cells, which explains the compromised affinity maturation in miR-155-KO mice^22,57,58^ (Fig. 7).

While *Mir155*-defcient mice show a compromised proliferation of GC-B cells, in vitro cultured KO B cells exhibit normal DNA synthesis and cell proliferation rates regardless of stimuli^57–59^. Thus, it was unclear why these in vitro cultured B cells cannot recapitulate the phenotype observed in vivo. In this study, we demonstrated that essential function of miR-155 is revealed under hypoxia in vitro and reproduced the known in vivo phenotypes observed in KO B cells. We have identified *Kdm2a* as a critical target gene of miR-155, which is induced under hypoxic conditions. *Kdm2a* exhibits context-dependent effects on proliferation^18,19^ and may act as a negative regulator of proliferation in GC-B cells. Therefore, miR-155-mediated *Kdm2a* repression enabled GC-B cells to initiate cell division under hypoxic conditions in the LZ during positive selection.

Our findings demonstrated the critical role of miR-155 in facilitating the metabolic adaptation of positively selected GC-B cells for cell division while mitigating hypoxia-driven stress in the LZ. Our previous studies have shown that high-affinity clones display greater proliferative activity than low-affinity clones within cMyc^+^ LZ GC-B cell subpopulation^7^. Indeed, miR-155 exerted its most potent suppressive effect on *Kdm2a* especially within the high-affinity clones of the cMyc^+^ LZ GC-B cell subpopulation (Fig. 3f). In agreement with this, *Mir155*-deficient mice exhibit a selective loss of high-affinity GC-B cells due to apoptosis^22,57,58^. In the absence of miR-155, cMyc^+^ LZ GC-B cells with higher levels of KDM2A experience detrimental effects such as increased ROS production due to dysfunctional mitochondrial and subsequent cell death (Fig. 1b, c, Fig. 6d, e). Despite the downregulation of miR-155 expression in cMyc^+^ DZ GC-B cells and its absence in cMyc^-^ DZ GC-B cells, there was a persistent increase in ROS production in these cells when miR-155 was absent. This may be because DZ GC-B cells prioritize energy use for mitosis rather than mitochondrial repair. Therefore, GC-B cells appear to be incapable of restoring mitochondrial function after migration to the DZ. The findings underscore the importance of the miR-155-*Kdm2a* regulatory axis in the LZ, which promoted metabolic reprogramming and gradual enhancement of OXPHOS in positively selected GC-B cells. As a result, positively selected GC-B cells could cope with the higher rate of cell division in the DZ by utilizing ATP provided by OXPHOS in remodeled mitochondria. These results are consistent with a previous observation that GC-B cells with higher affinity require increased OXPHOS activity to undergo extensive proliferation^9^.

KDM2A expression was upregulated > 1.5-fold in *Mir155*-KO mice compared to WT mice in both cMyc^+^ and cMyc^-^ DZ GC-B cells (Fig. 3e). However, the expression level of KDM2A in DZ GC-B cells of MUT mice was comparable to that of WT mice. Firstly, there is evidence for a 6-mer miR-155 binding site in the 3’UTR of mouse *Kdm2a*^60^. Since this 6-mer binding site is intact in MUT mice, miR-155 may partially repress *Kdm2a* transcripts through this region, inhibiting the KDM2A elevation in MUT mice. Alternatively, other miR-155 target(s) could contribute to the induction of KDM2A. *Hif1a* can be directly targeted by miR-155^60^ and potentially influences the expression of KDM2A because KDM2A may be induced partially in a HIF-1α-dependent manner under hypoxic conditions^41^. Given that *Hif1a* is repressed by miR-155 in a similar manner in both WT and MUT mice, the lower HIF-1α expression in MUT B cells compared to KO B cells may limit the efficiency of KDM2A induction in MUT mice.

MiRs generally have multiple targets. We identified 18 mRNAs that are predicted miR-155 targets in cMyc^+^ LZ GC-B cells and exhibit increased expression in the absence of miR-155 (Supplementary Fig. 3a). One of the molecules, *Jarid2* was previously discovered as a miR-155 target in positively selected GC-B cells, and its overexpression has been shown to increase apoptosis in LZ GC-B cells^22^. JARID2 functions as an accessory component of the Polycomb Repressive Complex 2 (PRC2), which catalyzes H3K27me3^61^, thereby epigenetically regulating gene expression. Therefore, the modulation of PRC2 activity by JARID2 potentially contributes to elevated apoptosis in these cells. Another molecule, F-Box Only Protein 11 (*Fbxo11*), was also among the identified 18 genes, and is a part of a multi-subunit E3 ubiquitin-ligase protein complex that catalyze the ubiquitination of proteins destined for proteasomal degradation. GC-B cell-specific deletion of *Fbxo11* increases the number of GC-B cells, particularly DZ GC-B cells, suggesting that this molecule is associated with GC-B cells proliferation^62^. Therefore, the phenotypic alterations observed in *Mir155*-deficient mice may result from the cumulative effects of several causal targets acting together with *Kdm2a*.

Collectively, our findings demonstrate that miR-155 acts as an epigenetic regulator to limit the activity of *Kdm2a* on target genes, thereby maintaining appropriate levels of H3K36me2. The precise control of H3K36me2 levels during positive selection is vital for the optimal expression of nuclear mitochondrial genes in GC-B cells under hypoxic conditions, thus facilitating mitochondrial remodeling. The resultant metabolic reprogramming is indispensable for the survival and robust proliferation of GC-B cells within the DZ. Our findings provide insights that could pave the way for the development of innovative therapeutics targeting histone PTMs to control GC responses in the context of vaccination and lymphomagenesis.

## Methods

### Mice

*Mir155*-deficient mice^59^, SW_HEL_ mice^23^, *Myc*^gfp/gfp^ mice^24^, *Aicda*-cre-hCD2^25^, µMT (JAX:002288, The Jackson laboratories) and B6.SJL CD45.1 (strain code:494, Charles River Laboratories) mice were maintained on C57BL/6 J background. SW_HEL_ mice were interbred with *Myc*^gfp/gfp^ and *Aicda*-cre-hCD2 mice and were maintained either *Mir155*-sufficient or *Mir155*-deficient background. B6.SJL CD45.1 mice were crossed with C57BL/6 J mice (JAX:000664, The Jackson laboratories), and heterozygous mice were used. In this study, male and female mice aged 6–25-weeks were used and euthanized by cervical dislocation. For the generation of *Kdm2a* mutant mice, a single guide RNA (AAATTAAATGTAAGCATTAA) was designed using CRISPOR^63^ to cut in the 3’UTR of the *Kdm2a* gene (Position 3369 – 3376 of *Kdm2a* 3’UTR, ENST00000398645.2). A 111 bp ssODN (CTTATTTAGAGTCGATCTCCAATGTTGTGCTAAGATTTTTAAATTAAATGTAAGatgcAtGGGAATGAGTCTTAGGCCACCAGCTGACACATTGGCATTATTTTGGGTCAG) was designed containing the “mir155-2” mutation. Zygotes were generated by superovulation of C57BL/6 J mice and electroporated with 50 µL of Cas9 protein / sgRNA / ssODN (final concentrations 1.2 µM / 6 µM / 8 µM respectively). Both template and guide were added at the 1 cell stage. Electroporation of the embryos was performed using a Nepa21 electroporator (Sonidel). Two males (1 heterozygous and 1 homozygous) were found to have sequence perfect integration of the mir155-2 mutation via Sanger sequencing (Forward primer: CGAGGACGTAGAACATGCGA, Reverse primer: GGGCAGGCACTTCCTAGTTT) and were further bred to C57BL/6 J yielding 2 heterozygous F1s. Q-PCR CNV analysis further confirmed the presence of only one copy of the mir155-2 mutation. F1 mice were inbred to generate homozygous mutant mice. All mice were bred and kept under specific-pathogen-free conditions at the Francis Crick Institute Biological Research Facility in accordance with guidelines set by UK Home Office and the Francis Crick Institute Ethical Review Panel.

### Immunization, treatments, and adoptive transfers

Mice were immunized intraperitoneally with 2 × 10^8^ SRBCs. For HEL-specific GC responses, splenic cells from donor mice were incubated with biotinylated antibodies: anti-CD43, anti-CD4 and anti-Ter-119, washed and incubated with anti-biotin microbeads (Miltenyi Biotec). B cells were enriched by biotin negative selection using MACS LS Columns (Miltenyi Biotec). The purity of recovered CD19^+^ B220^+^ B cells was > 97%. 5 × 10^4^ to 1 × 10^5^ HEL-specific B cells were transferred into B6.SJL CD45.1^+^ hosts intravenously. Mice were immunized intraperitoneally on the same day of cell transfer or one day later with 2 × 10^8^ SRBCs conjugated with HEL^3 ×^ . The HyHEL10 BCR of SW_HEL_ mice exhibits a binding affinity 13,000-fold lower to HEL^3 ×^ than to HEL. Immunization with HEL^3 ×^ induces robust and persistent GC responses at the expense of extrafollicular responses^64^. HEL^3 ×^ protein was harvested from the supernatant of Chinese hamster ovary (CHO) cells expressing his-tagged HEL^3 ×^ ^65^ and was purified using a His-Trap FF column (Cytiva) on AKTA Pure column chromatography system (Cytiva). Eluted protein was concentrated to 0.5 ml (Vivaspin 15, 10,000 Da cutoff concentrator) and applied to a S200 10/300 analytical size exclusion column (Cytiva). HEL^3 ×^ was found to eluted as a single, symmetrical peak and snap frozen at 1 mg/ml and stored at -80 °C until required. For B cell transfer from wild type (*Kdm2a*-mutant littermates) or *Kdm2a*-mutant mice, splenic B cells from donor mice were enriched by negative selection using MACS LS Columns as described above. 2.5 × 10^7^ to 3 × 10^7^ B cells were transferred into µMT hosts intravenously. Mice were immunized intraperitoneally on the same day of cell transfer with 2 × 10^8^ SRBCs. To detect hypoxia, the hypoxia dye MAR was used. MAR is non-fluorescent compound that is irreversibly converted into a fluorescent molecule by a reductase that is activated in response to low oxygen concentrations ( ~ 5% O_2_)^44^. The conversion of MAR only occurs under hypoxic conditions^44^, and the fluorescence of MAR is not affected by increased oxygen concentrations during tissue processing. Eight days after subcutaneous immunization of C57BL/6 mice with 50 μg of 4-Hydroxy-3-nitrophenyl acetyl hapten conjugated chicken gamma globulin (NP-CGG) in Imject Alum Adjuvant (ThermoFisher Scientific), 4.3 µg MAR hypoxia detecting probe (Goryo Chemical) in PBS per site or control DMSO in PBS was subcutaneously injected into two sites around the immunized inguinal lymph node. Three hours later, lymph nodes were harvested for flow cytometric analysis. Non-GC-B cells were gated as CD19^+^ B220^+^ CD38^+^ CD138^-^ CD95^-^ GL7^-^.

### Flow cytometry and cell sorting

Multicolor flow cytometry for analyses or for cell sorting was performed on LSR Fortessa or FACSAria (BD Biosciences) and data were analyzed using FlowJo v.10.8.1 software. PBS supplemented with 2% FBS was used as flow cytometry buffer. Dead cells were excluded using Zombie Fixable viability kit (BioLegend) during cell sorting. Harvested splenic B cells were enriched by CD43, Ter119 and CD4 biotin negative selection using MACS LS Columns to remove CD43^+^ plasmablasts and non-B cells for analysis. For HEL-specific GC responses, splenic B cells were enriched by CD45.1 negative selection using MACS LS Columns to remove recipient-derived cells. Single-cell suspensions were prepared and stained for the antibodies specific for cell surface markers. HEL-binding cells were stained with HEL (50 ng/ml), followed by HyHEL9 Alexa Fluor 647^64^. For active-caspase 3 staining, after surface marker staining, cells were fixed and permeabilized using a Fixation/Permeabilization Solution Kit (BD Biosciences) and stained with anti-active caspase 3 antibody (BD Biosciences). For KDM2A and H3K36me2 staining, cells were stained for surface marker antibodies and were fixed and permeabilized using a True-Nuclear Transcription Factor Buffer Set (BioLegend). The permeabilized cells were stained with anti-KDM2A or anti-H3K36me2antibody, followed by donkey anti-rabbit antibody staining. Cells stained with an anti-rabbit antibody, but no primary antibodies determined the basal level of fluorescence intensity for KDM2A/H3K36me2, and the basal fluorescence intensity was subtracted from each MFI of KDM2A/H3K36me2 staining for the calculation of relative MFI. For MitoSOX detection, cells were stained for surface marker antibodies. Then, they were incubated in HBSS with Calcium and Magnesium (ThermoFisher scientific) supplemented with 2.5 µM MitoSOX Red (ThermoFisher Scientific) and 50 nM Mito Tracker Deep Red FM (ThermoFisher Scientific) for 20 - 30 min at 37 °C and washed as described by the manufacturer before analysis.

### Antibodies

CD43, biotin (S7), BD Biosciences, 553269; CD4, biotin (GK1.5), BioLegend, 100404; Ter119, biotin (TER-119), BioLegend, 116204; CD45.1, biotin (A20), ThermoFisher Sciences, 13-0453-85; CD21/35, BV786 (7G6), BD Biosciences, 740894; CD23, FITC (B3B4), BD Biosciences, 553138; CD93, PE (AA4.1), ThermoFisher Sciences, 12-5892-82; CXCR4, PerCP-eFluor710 (2B11), ThermoFisher Sciences, 46-9991-82; CXCR4, APC (2B11), BD Biosciences, 558644; CD86, FITC (GL1), BD Biosciences, 553691; CD86, PECy7 (GL1), BioLegend, 105014; CD86, APCCy7 (GL1), BioLegend, 105030; CD86, BV605 (GL1), BioLegend, 105037; CD86, BV785 (GL1), BioLegend, 105043; CD95, PECy7 (Jo2), BD Biosciences, 557653; CD95, APC-R700 (Jo2), BD Biosciences, 565130; CD95, BV786 (Jo2), BD Biosciences, 740906; CD38, PerCP-eFluor710 (90), ThermoFisher Sciences, 46-0381-82; CD38, PECy7 (90), BioLegend, 102718; CD38, BV650 (90), BD Biosciences, 740489; CD38, BUV395 (90), BD Biosciences, 740245; GL7, Pacific blue (GL7), BioLegend, 144614; GL7, Alexa Fluor 488 (GL7), ThermoFisher Sciences, 53-5902-82; human CD2, PECy7 (TS1/8), BioLegend, 309214; human CD2, APC (TS1/8), BioLegend, 309224; human CD2, Alexa700 (TS1/8), BioLegend, 309228; CD45.2, BV605 (104), BD Biosciences, 563051; CD45.2, BV786 (104), BD Biosciences, 563686; CD45.2, BUV395 (104), BD Biosciences, 564616; CD138, PE, (281-2), BioLegend, 142504; CD138, APC, (281-2), BioLegend, 142506; CD138, APCCy7, (281-2), BioLegend, 142530; CD138, BV605, (281-2), BioLegend, 142531; CD138, BV786, (281-2), BD Biosciences, 740880; B220, FITC (RA3-6B2), ThermoFisher Sciences, 11-0452-82; B220, APCCy7 (RA3-6B2), ThermoFisher Sciences, 47-0452-82; B220, BV421 (RA3-6B2), BioLegend, 103240; B220, BV650 (RA3-6B2), BioLegend, 103241; B220, BUV787 (RA3-6B2), BD Biosciences, 612838; CD19, FITC (6D5), BioLegend, 115506; CD19, BV605 (6D5), BioLegend, 115540; CD19, BV650 (6D5), BioLegend, 115541; CD19, PE-CF594 (1D3), BD Biosciences, 562291; IgM, BV605 (II/41), BD Biosciences, 743325; IgD, Alexa Fluor 647 (11-26 c.2a), BioLegend, 405708; IgG1, FITC (A85-1), BD Biosciences, 553443; IgG1, CF-594 (A85-1), BD Biosciences, 562559; Igκ light chain, BV421 (187.1), BD Biosciences, 562888; rabbit IgG, Alexa Fluor 647 (Poly4064), BioLegend, 406414; rabbit IgG, PE (Poly4064), BioLegend, 406421; rabbit IgG, Alexa Fluor 488 (Poly4064), BioLegend, 406416; CD40, purified (HM-40), BioLegend, 102902; active caspase 3, PE (C92-605), BD Biosciences, 550821; KDM2A, purified (EPR18602), Abcam, ab191387; cMYC, Alexa Fluor 555 (9E10), ThermoFisher Sciences, MA1-980-A555; Puromycin, Alexa Fluor 488 (12D10), Merck, MABE343-AF488; Histone H3K36me2, purified (EPR16994(2)), Abcam, ab176921; mouse IgM F(ab’)_2_ fragment, Jackson ImmunoResearch, 115-006-020; Streptavidin, PerCP-Cy5.5, BioLegend, 405214; Streptavidin, PE, ThermoFisher Sciences, 12-4317-87; Streptavidin, BV650, BioLegend, 405231; Streptavidin, BUV737, BD Biosciences, 612775.

### B cell culture, proliferation assays, ATP measurement and SCENITH

Harvested splenic B cells from *Mir155*-sufficient or *Mir155*-deficient C57BL/6 mice were enriched by CD43, Ter119 and CD4 biotin negative selection using MACS LS Columns. Purified B cells were cultured with positive selection mimicking stimulants (1 µg/ml anti-CD40 antibody (HM40-3), 5 µg/ml AffiniPure F(ab’)_2_ Fragment Goat Anti-Mouse IgM (H + L) (Jackson ImmunoResearch) and 4 ng/ml murine IL-4 (PeproTech) in B cell medium (RPMI 1640 GlutaMAX Supplement medium supplemented with 10 % fetal bovine serum, 1 % pen-strep solution and 50 µM β-mercaptoethanol) at 37 °C in a humidified chamber containing 5% CO_2_ and either 21% O_2_ (normoxia) or 1% O_2_ (hypoxia). For CTV proliferation assays, purified B cells were stained with 5μM CellTrace Violet (ThermoFisher Scientific) in PBS for 15 min at RT and washed as described by the manufacturer. For ATP measurement, purified B cells were cultured for 2 days as described above. On the measurement day, cells were washed twice in PBS to remove glucose and resuspended in either glucose medium (DMEM no glucose medium supplemented with 1 % pen-strep solution, 50 µM β-mercaptoethanol and 10 mM D-(+)-glucose) or galactose medium (DMEM no glucose medium supplemented with 1 % pen-strep solution, 50 µM β-mercaptoethanol and 10 mM galactose). After a thorough wash to remove glucose in the medium, 1 × 10^5^ cells were plated per well and were further cultured in the presence or absence of 5 µM oligomycin A (Merck) at 37 °C in a humidified chamber containing 5% CO_2_ and 1% O_2_ for 2 h. All the cells were cultured in hypoxic conditions for 2 h to directly compare ATP production ability under the same condition. ATP was measured with Mitochondrial ToxGlo Assay kit (Promega) and followed the manufacturer’s protocol. SCENITH was performed as previously described^33^ with modifications. Splenic B cells derived from SW_HEL_ adoptive transfer were first stained for surface marker antibodies, then divided into four groups and incubated for 30 min at 37 °C in B cell medium. Metabolic inhibitors (1 µM Oligomycin and/or 2-Deoxy-D-Glucose (2-DG) at the final concentration) or DMSO were then added, followed by a 15 min incubation at 37 °C. Puromycin (10 µg/ml final concentration) was directly added, and cells were incubated for an additional 15 min at 37 °C. Cells were washed and fixed using BD Cytofix/Cytoperm solution. Fixed cells were then stained intracellularly with anti-Puromycin antibody and washed immediately before flow cytometric analysis. All chemicals used for SCENITH were purchased from Merck. Glucose dependence and FAO/AAO (fatty acid oxidation and amino acid oxidation) capacity were calculated using the gMFI values of anti-Puromycin staining with the following formulas: Glucose dependence (%) = 100 × [(DMSO – 2DG) / (DMSO-2-DG & Oligomycin)], FAO/AAO capacity (%) = 100 – {100 × [(DMSO – 2DG) / (DMSO-2-DG & Oligomycin)]}.

### Immunofluorescence

Harvested spleens were embedded in OCT compound. 9 μm cryosections were fixed in ice cold acetone for 3 min, followed by air dry for 20 min. The sections were blocked with blocking buffer (0.3% Triton X-100, 1% BSA, 5% normal bovine serum and 1% rabbit serum in PBS) and stained with the described antibodies for overnight at 4 C°. For MitoTracker staining of cultured B cells, purified B cells were cultured for 2 days as described above and washed in PBS twice. Cell suspension containing 2 − 3 × 10^6^ cells/ml in PBS was placed on Shi-fix coverslips (Shikhar Biotech) and incubated at 37 °C in a humidified chamber containing 5% CO_2_ and 1% O_2_ for 30–40 min. The coverslips were then washed as instructed by the manufacturer. Cells bound on the coverslips were stained with 83 nM MitoTracker Orange CM-H_2_TMRos (ThermoFisher Scientific) and 50 nM Mito Tracker Deep Red FM at 37 °C for 15 min, followed by wash in warm PBS and fixation with 4% formaldehyde (ThermoFisher Scientific). Slides were mounted with Prolong Gold Antifade Reagent with DAPI (ThermoFisher Scientific), and images were acquired using LSM880 inverted confocal microscope (Zeiss) with 63 × objective and Zen Microscopy software (Zeiss).

### Image analysis

For the detection of mitochondrial ROS on confocal images of in vitro cultured B cells, the ratiometric cytoplasmic-to-nuclear intensity of mitochondrial ROS was quantified using a custom-built CellProfiler (v.4.2.5)^66^ pipeline. Uneven illumination was first corrected in the Mito Tracker Deep Red FM and MitoTracker Orange CMTMRos channels by dividing the pixel values by a polynomial fit to the intensity of the image pixels. Following smoothing with median filtering, objects were then detected in the DAPI and Mito Tracker Deep Red FM channels using minimum cross-entropy (Mito Tracker Deep Red FM) or Otsu (DAPI) thresholding. All objects detected in the DAPI channel are then associated with corresponding objects in the Mito Tracker Deep Red FM channel, i.e., any Mito Tracker Deep Red FM objects without any corresponding DAPI objects are removed. The cytoplasm was then defined as the area within Mito Tracker Deep Red FM objects not occupied by DAPI objects. The mean intensity in the MitoTracker Orange CMTMRos channel was then measured within each cytoplasm object, and this was divided by the background value, the mean intensity in the MitoTracker Orange CMTMRos channel of each DAPI object to minimize batch effects. Cells with incorrect segmentation and apoptotic cells in the images were manually removed. For the analysis of co-expression of H3K36me2 / MYC and MYC / KDM2A in spleen sections was quantified using a custom FIJI script^67^. First, the approximate centers of cells were detected by identifying local extrema in eigenvalues of the Hessian tensor, calculated based on the IgD channel using FeatureJ (https://imagescience.org/meijering/software/featurej/hessian/). Cells were then fully segmented using these detected centers of cells as “markers” in a marker-controlled watershed operation, implementing using MorphoLibJ^68^, using a Gaussian- then Sobel-filtered version of the IgD channel as input. The mean intensity of H3K36me2, MYC or KDM2A within the segmented cells was then quantified using ImageJ FIJI (v.2.9.0). All cells outside a manually defined region of interest were excluded.

### Analysis of histone PTMs

Cells were prepared for sorting by flow cytometry as described above. LZ or DZ GC-B cells ( > 1 × 10^5^) were directly sorted into 2 N H_2_SO_4_ as previously described^35^. After a 30 min incubation at room temperature, cellular debris was removed by centrifugation at 4000 × g for 5 min and histones were precipitated from the supernatant with trichloroacetic acid at a final concentration of 20% (v/v) overnight at 4 °C. Precipitated histones were pelleted with centrifugation at 10,000 × g for 5 min at 4 °C, washed once with 0.1% HCl in acetone, then once with 100% acetone with centrifugation at 15,000 × g for 5 min at 4 °C. Histones were briefly dried with SpeedVac concentrator (Eppendorf) and stored at -80 °C until derivatization. Histones were propionylated and digested according to Garcia et al.^36^ with the modification of a single round of propionylation for 1 hr prior to and following digestion. Targeted LC-MS/MS was performed on a Thermo TSQ Quantiva (ThermoFisher Scientific). All injections were performed in technical triplicate. Raw data were analyzed with Skyline (v.21.2)^69^ according to published methods^35^. The relative abundance of each histone PTM was calculated from the total peak areas exported from Skyline. All the chemicals were purchased from ThermoFisher Sceintific.

### RNA-seq and data analysis

*Mir155*-sufficient or *Mir155*-deficient cMyc-GFP^+^ LZ GC-B cells were sorted by flow cytometry 5 days after HEL^3 ×^ -SRBC immunization. Total RNA was prepared with TRIzol Reagent (ThermoFisher Scientific) combined with RNeasy Mini Kit (QIAGEN) and processed using a TruSeq Stranded mRNA Sample Prep kit (Illumina). RNA-sequencing was carried out on the Illumina HiSeq 2500 platform and generated ~43 million, 75 bp paired-end reads per sample. The RSEM package (v.1.2.31)^70^ in conjunction with the bowtie2 alignment algorithm (v.2.2.9)^71^ was used for the mapping and subsequent gene-level counting of the sequenced reads with respect to Ensembl mouse GRCm.38.86 version transcriptome. Normalization of raw count data and differential expression analysis was performed with the DESeq2 package (v.1.24.1)^72^ within the R programming environment (v.3.6.0)^73^. Differentially expressed genes were defined as those showing statistically significant differences between KO and WT sample groups (FDR < 0.05). Majority of Mir155-deficient donor mice were males, whereas majority of Mir155-sufficient donor mice were female. Thus, the following 4 genes that are in the Y chromosome and more enriched in KO than WT mice were removed from the list of DEGs (ENSMUSG00000069045, ENSMUSG00000069049, ENSMUSG00000056673 and ENSMUSG00000068457). Gene list ranked by the Wald statistic were used to look for pathway and biological process enrichment using the Broad’s GSEA software (v.2.1.0) with gene sets from MSigDB (v6)^74^. GSEA results were visualized with EnrichmentMap (v3.3.4)^75^ in Cytoscape (v.3.7). We employed three miR target prediction tools, MiRDB^76^, Diana^77^ and Target scan^78^ for identifying miR-155 targets.

### scRNA-seq and data analysis

Donor-specific *Mir155*-sufficient or *Mir155*-deficient GC-B cells were sorted 7 days after HEL^3 ×^ -SRBC immunization. Libraries were prepared with Chromium Next GEM Single Cell 5’ Reagent Kit v2 (10 × Genomics) and sequenced on the Illumina NovaSeq 6000 platform. Fastq files were aligned and counted using CellRanger (v.6.1.2) and using as reference mm10-2020-A (obtained from 10 × Genomics). Matrices were imported into Seurat (v.4.0.5)^79^, which was sued for downstream analysis. After QC, cells with less than 1000 features and more than 10% mitochondrial content were removed. Data was then normalized, scaled and variable features were identified per cell. Samples were integrated using “CCA” by integrating in the following order, first all replicates for each genotype and then by genotypes. DoubletFinder^80^ was used to predict doublets. Clusters identified as contaminating for expression of Cd8a, Cd3e, Cd3d, Lyz2 and Gzma, and cells labelled as doublets were removed from the analysis. Clusters were manually annotated using differential expressed genes obtained from FindMarkers. Uniform Manifold Approximation and Projection (UMAP) was used for visualization. VlnPlots and FeaturePlots have been used to display gene expression values per cluster or overlay on UMAP. The merged dataset of biological triplicates contained 25,358 and 24,087 GC-B cells for WT and KO cells, respectively. Unsupervised clustering using Seurat identified nine distinct clusters, each with their unique cell cycle status determined by the expression of genes associated with different phases of the cell cycle (Supplementary Fig. 1g). For gene signatures, a score was calculated using AddModuleScore prior to plotting. Statistical significance was obtained using Wilcoxon test and multiple comparison correction was performed using the “fdr” procedure. Differential gene expression between genotypes per cluster was carried out using a pseudobulk approach, in which gene counts were aggregated per sample and the “pseudoBulkDGE” function from the scran package^81^ was used with the design ~genotype. Genes are considered differentially expressed if FDR < 0.05. For trajectory analysis, Partition-based graph abstraction PAGA^29^ was used. The integrated Seurat object was converted into a h5 format, neighbors were computed using 20 pcs and clusters detected using Seurat were used to compute the PAGA graph. Mitochondrial gene list was downloaded from MitoCarta3.0^55^.

### ChIP-seq and data analysis

WT and KO B cells were cultured under hypoxic conditions with positive selection mimicking stimulants for 2 days. Cells were fixed for 1 hr in 2 mM EGS, followed by 15 min in 1% Formaldehyde. Chromatin was sheared using Bioruptor Pico sonication device (Diagenode). An aliquot of the chromatin was used to assess the size of the DNA fragments obtained by High Sensitivity NGS Fragment Analysis Kit (Agilent) on Fragment Analyzer (Agilent). ChIP was performed using IP-Star Compact Automated System (Diagenode). Chromatin corresponding to 13.5 µg and 1 µg were immunoprecipitated with KDM2A antibody (a gift from R. Klose, University of Oxford) and H3K36me2 (clone: EPR18602) (Abcam). Chromatin corresponding to 1% was used as Input. Libraries were prepared using MicroPlex Library Preparation Kit v3 (Diagenode) with 24 UDI for MicroPlex v3 (Diagenode). Generated libraries were purified using Agencourt AMPure XP (Beckman Coulter) and quantified using Qubit 1 × dsDNA HS Assay Kit (ThermoFisher Scientific). The fragment size was analyzed by High Sensitivity NGS Fragment Analysis Kit on Fragment Analyzer. Sequencing was carried out on the Illumina NovaSeq 6000 platform. Read alignment, library QC, peak detection and quantification was carried out using the nf-core/chipseq pipeline (v.1.2.2), using “-profile crick --min_reps_consensus 2”. Briefly, reads were aligned using BWA^82^ to GRCm38 using the genomes provided with the pipeline. Peaks were called using MACS2^83^ and annotated using HOMER^84^. A list of consensus peaks is generated as the union of peaks detected in at least two replicates per condition. Bigwigs generated by the pipeline (scaled to 1 million mapped reads) were used to generate meta profile plots with Deeptools “compute matrix”^85^. A table containing CpG island containing genes was obtained from UCSC Table Browser^86^. Initially, an average of 26436 and 17586 KDM2A peaks were identified for WT and KO cells, respectively. The peaks that only one of the triplicate samples bound were removed from the subsequent analysis. After the exclusion of peaks, there were 9129 KDM2A peaks that were bound by both WT and KO B cells. The average fold change (fc) was calculated by dividing the average KDM2A peak signal by the input signal, and then “WT-enriched” or “KO-enriched” peaks among the 9129 KDM2A peaks bound by both WT and KO B cells were determined based on a fold change threshold (fc > 3) and a ratio of average fc between WT and KO cells ( > 2^0.23^-fold enrichment). Peaks with an fc > 3 from “peaks bound only by WT” (5905) and “peaks bound only by KO” (1479) were also categorized as “WT-enriched” and “KO-enriched” KDM2A peaks, respectively. After applying the selection threshold, a total of 1403 genes with 1453 “WT-enriched KDM2A” peaks and 309 genes with 309 “KO-enriched KDM2A” peaks were identified (Supplementary Data 5). Among these, 73.6% and 78.0% of the “WT-enriched KDM2A” peaks and “KO-enriched KDM2A” peaks, respectively are genes containing CpG island.

### Statistical analysis

Statistical significance was determined by unpaired, two-tailed Student’s t-test, Kruskal-Wallis one-way analysis of variance (ANOVA) with multiple comparisons, Wilcoxon rank sum test, or Kolmogorov-Smirnov test. The statistical method used is indicated in the respective Figure legends. Prism (v.9.4.1) software (GraphPad) and R were used to calculate all statistical analyses.

### Reporting summary

Further information on research design is available in the Nature Portfolio Reporting Summary linked to this article.

## Supplementary information

Supplementary Information

Description of Additional Supplementary Files

Supplementary Data 1

Supplementary Data 2

Supplementary Data 3

Supplementary Data 4

Supplementary Data 5

Reporting Summary

Transparent Peer Review file

## Source data

Source Data

## Data Availability

The scRNA-seq, bulk RNA-seq and ChIP-seq data generated in this study have been deposited in the NCBU Gene Expression Omnibus (GEO) database under accession number GSE230528, GSE231649, and GSE230527, respectively. Source data are provided with this paper and can be accessed at the following links: https://www.ncbi.nlm.nih.gov/geo/query/acc.cgi?acc=GSE230528, https://www.ncbi.nlm.nih.gov/geo/query/acc.cgi?acc=GSE231649, https://www.ncbi.nlm.nih.gov/geo/query/acc.cgi?acc=GSE230527. Source data are provided with this paper.

## References

[1] Berek, C., Berger, A. & Apel, M. Maturation of the immune response in germinal centers. *Cell* **67**, 1121–1129 (1991). 1760840 10.1016/0092-8674(91)90289-b

[2] Jacob, J., Kelsoe, G., Rajewsky, K. & Weiss, U. Intraclonal generation of antibody mutants in germinal centres. *Nature* **354**, 389–392 (1991). 1956400 10.1038/354389a0

[3] Kepler, T. B. & Perelson, A. S. Cyclic re-entry of germinal center B cells and the efficiency of affinity maturation. *Immunol. Today* **14**, 412–415 (1993). 8397781 10.1016/0167-5699(93)90145-B

[4] Nakagawa, R. & Calado, D. P. Positive Selection in the Light Zone of Germinal Centers. *Front Immunol.* **12**, 661678 (2021). 33868314 10.3389/fimmu.2021.661678 PMC8044421

[5] Calado, D. P. et al. The cell-cycle regulator c-Myc is essential for the formation and maintenance of germinal centers. *Nat. Immunol.* **13**, 1092–1100 (2012). 23001146 10.1038/ni.2418 PMC4132664

[6] Dominguez-Sola, D. et al. The proto-oncogene MYC is required for selection in the germinal center and cyclic reentry. *Nat. Immunol.* **13**, 1083–1091 (2012). 23001145 10.1038/ni.2428 PMC3711534

[7] Nakagawa, R. et al. Permissive selection followed by affinity-based proliferation of GC light zone B cells dictates cell fate and ensures clonal breadth. *Proc. Natl Acad. Sci. USA* **118**, e2016425118 (2021). 33419925 10.1073/pnas.2016425118 PMC7812803

[8] Weisel, F. J. et al. Germinal center B cells selectively oxidize fatty acids for energy while conducting minimal glycolysis. *Nat. Immunol.* **21**, 331–342 (2020). 32066950 10.1038/s41590-020-0598-4 PMC7112716

[9] Chen, D. et al. Coupled analysis of transcriptome and BCR mutations reveals role of OXPHOS in affinity maturation. *Nat. Immunol.* **22**, 904–913 (2021). 34031613 10.1038/s41590-021-00936-y

[10] Jellusova, J. et al. Gsk3 is a metabolic checkpoint regulator in B cells. *Nat. Immunol.* **18**, 303–312 (2017). 28114292 10.1038/ni.3664 PMC5310963

[11] Cho, S. H. et al. Germinal centre hypoxia and regulation of antibody qualities by a hypoxia response system. *Nature* **537**, 234–238 (2016). 27501247 10.1038/nature19334 PMC5161594

[12] Kierans, S. J. & Taylor, C. T. Regulation of glycolysis by the hypoxia-inducible factor (HIF): implications for cellular physiology. *J. Physiol.* **599**, 23–37 (2021). 33006160 10.1113/JP280572

[13] Goda, N. et al. Hypoxia-inducible factor 1alpha is essential for cell cycle arrest during hypoxia. *Mol. Cell Biol.* **23**, 359–369 (2003). 12482987 10.1128/MCB.23.1.359-369.2003 PMC140666

[14] Koshiji, M. et al. HIF-1alpha induces cell cycle arrest by functionally counteracting Myc. *EMBO J.* **23**, 1949–1956 (2004). 15071503 10.1038/sj.emboj.7600196 PMC404317

[15] Liu, L. et al. Hypoxia-induced energy stress regulates mRNA translation and cell growth. *Mol. Cell* **21**, 521–531 (2006). 16483933 10.1016/j.molcel.2006.01.010 PMC3153113

[16] Abbott, R. K. et al. Germinal center hypoxia potentiates immunoglobulin class switch recombination. *J. Immunol.* **197**, 4014–4020 (2016). 27798169 10.4049/jimmunol.1601401 PMC5123804

[17] Li, L. et al. Regulation of humoral immune response by HIF-1alpha-dependent metabolic reprogramming of the germinal center reaction. *Cell Immunol.* **367**, 104409 (2021). 34246872 10.1016/j.cellimm.2021.104409

[18] Kawakami, E., Tokunaga, A., Ozawa, M., Sakamoto, R. & Yoshida, N. The histone demethylase Fbxl11/Kdm2a plays an essential role in embryonic development by repressing cell-cycle regulators. *Mech. Dev.* **135**, 31–42 (2015). 25463925 10.1016/j.mod.2014.10.001

[19] Tanaka, Y. et al. JmjC enzyme KDM2A is a regulator of rRNA transcription in response to starvation. *EMBO J.* **29**, 1510–1522 (2010). 20379134 10.1038/emboj.2010.56 PMC2876952

[20] Esposito, L. A., Melov, S., Panov, A., Cottrell, B. A. & Wallace, D. C. Mitochondrial disease in mouse results in increased oxidative stress. *Proc. Natl Acad. Sci. USA* **96**, 4820–4825 (1999). 10220377 10.1073/pnas.96.9.4820 PMC21775

[21] Redza-Dutordoir, M. & Averill-Bates, D. A. Activation of apoptosis signalling pathways by reactive oxygen species. *Biochim Biophys. Acta* **1863**, 2977–2992 (2016). 27646922 10.1016/j.bbamcr.2016.09.012

[22] Nakagawa, R. et al. MicroRNA-155 controls affinity-based selection by protecting c-MYC+ B cells from apoptosis. *J. Clin. Invest* **126**, 377–388 (2016). 26657861 10.1172/JCI82914 PMC4701536

[23] Phan, T. G. et al. B cell receptor-independent stimuli trigger immunoglobulin (Ig) class switch recombination and production of IgG autoantibodies by anergic self-reactive B cells. *J. Exp. Med* **197**, 845–860 (2003). 12668643 10.1084/jem.20022144 PMC2193892

[24] Huang, C. Y., Bredemeyer, A. L., Walker, L. M., Bassing, C. H. & Sleckman, B. P. Dynamic regulation of c-Myc proto-oncogene expression during lymphocyte development revealed by a GFP-c-Myc knock-in mouse. *Eur. J. Immunol.* **38**, 342–349 (2008). 18196519 10.1002/eji.200737972

[25] Kwon, K. et al. Instructive role of the transcription factor E2A in early B lymphopoiesis and germinal center B cell development. *Immunity* **28**, 751–762 (2008). 18538592 10.1016/j.immuni.2008.04.014

[26] Laidlaw, B. J., Duan, L., Xu, Y., Vazquez, S. E. & Cyster, J. G. The transcription factor Hhex cooperates with the corepressor Tle3 to promote memory B cell development. *Nat. Immunol.* **21**, 1082–1093 (2020). 32601467 10.1038/s41590-020-0713-6 PMC7442689

[27] Suan, D. et al. CCR6 defines memory B cell precursors in mouse and human germinal centers, revealing light-zone location and predominant low antigen affinity. *Immunity* **47**, 1142–1153.e1144 (2017). 29262350 10.1016/j.immuni.2017.11.022

[28] La Manno, G. et al. RNA velocity of single cells. *Nature* **560**, 494–498 (2018). 30089906 10.1038/s41586-018-0414-6 PMC6130801

[29] Wolf, F. A. et al. PAGA: graph abstraction reconciles clustering with trajectory inference through a topology preserving map of single cells. *Genome Biol.* **20**, 59 (2019). 30890159 10.1186/s13059-019-1663-x PMC6425583

[30] Mroczek, S. & Kufel, J. Apoptotic signals induce specific degradation of ribosomal RNA in yeast. *Nucleic Acids Res* **36**, 2874–2888 (2008). 18385160 10.1093/nar/gkm1100 PMC2396418

[31] Toboso-Navasa, A. et al. Restriction of memory B cell differentiation at the germinal center B cell positive selection stage. *J. Exp. Med* **217**, e20191933 (2020). 32407433 10.1084/jem.20191933 PMC7336312

[32] Huang, G., Li, H. & Zhang, H. Abnormal expression of mitochondrial ribosomal proteins and their encoding genes with cell apoptosis and diseases. *Int J. Mol. Sci.* **21**, 8879 (2020). 33238645 10.3390/ijms21228879 PMC7700125

[33] Arguello, R. J. et al. SCENITH: a flow cytometry-based method to functionally profile energy metabolism with single-cell resolution. *Cell Metab.* **32**, 1063–1075 e1067 (2020). 33264598 10.1016/j.cmet.2020.11.007 PMC8407169

[34] Yazicioglu, Y. F. et al. Dynamic mitochondrial transcription and translation in B cells control germinal center entry and lymphomagenesis. *Nat. Immunol.* **24**, 991–1006 (2023). 37095377 10.1038/s41590-023-01484-3 PMC10232359

[35] Camarillo, J. M. et al. Coupling fluorescence-activated cell sorting and targeted analysis of histone modification profiles in primary human leukocytes. *J. Am. Soc. Mass Spectrom.* **30**, 2526–2534 (2019). 31286445 10.1007/s13361-019-02255-x PMC6917871

[36] Garcia, B. A. et al. Chemical derivatization of histones for facilitated analysis by mass spectrometry. *Nat. Protoc.* **2**, 933–938 (2007). 17446892 10.1038/nprot.2007.106 PMC4627699

[37] Zhao, Y. & Garcia, B. A. Comprehensive catalog of currently documented histone modifications. *Cold Spring Harb. Perspect. Biol.* **7**, a025064 (2015). 26330523 10.1101/cshperspect.a025064 PMC4563710

[38] Klein, B. J. et al. Histone H3K23-specific acetylation by MORF is coupled to H3K14 acylation. *Nat. Commun.* **10**, 4724 (2019). 31624313 10.1038/s41467-019-12551-5 PMC6797804

[39] Chen, H. et al. H3K36 dimethylation shapes the epigenetic interaction landscape by directing repressive chromatin modifications in embryonic stem cells. *Genome Res* **32**, 825–837 (2022). 35396277 10.1101/gr.276383.121 PMC9104706

[40] Tian, X. et al. Histone lysine-specific methyltransferases and demethylases in carcinogenesis: new targets for cancer therapy and prevention. *Curr. Cancer Drug Targets* **13**, 558–579 (2013). 23713993 10.2174/1568009611313050007 PMC3703250

[41] Batie, M., Druker, J., D’Ignazio, L. & Rocha, S. KDM2 Family Members are Regulated by HIF-1 in Hypoxia. *Cells* **6**, 8 (2017). 28304334 10.3390/cells6010008 PMC5371873

[42] Xia, X. et al. Integrative analysis of HIF binding and transactivation reveals its role in maintaining histone methylation homeostasis. *Proc. Natl Acad. Sci. USA* **106**, 4260–4265 (2009). 19255431 10.1073/pnas.0810067106 PMC2657396

[43] Zaidi, M., Fu, F., Cojocari, D., McKee, T. D. & Wouters, B. G. Quantitative visualization of hypoxia and proliferation gradients within histological tissue sections. *Front Bioeng. Biotechnol.* **7**, 397 (2019). 31867322 10.3389/fbioe.2019.00397 PMC6906162

[44] Piao, W. et al. Development of azo-based fluorescent probes to detect different levels of hypoxia. *Angew. Chem. Int Ed. Engl.* **52**, 13028–13032 (2013). 24127124 10.1002/anie.201305784

[45] Gross, M. W., Karbach, U., Groebe, K., Franko, A. J. & Mueller-Klieser, W. Calibration of misonidazole labeling by simultaneous measurement of oxygen tension and labeling density in multicellular spheroids. *Int J. Cancer* **61**, 567–573 (1995). 7759162 10.1002/ijc.2910610422

[46] Bruning, U. et al. MicroRNA-155 promotes resolution of hypoxia-inducible factor 1alpha activity during prolonged hypoxia. *Mol. Cell Biol.* **31**, 4087–4096 (2011). 21807897 10.1128/MCB.01276-10 PMC3187364

[47] Wan, G. et al. Hypoxia-induced MIR155 is a potent autophagy inducer by targeting multiple players in the MTOR pathway. *Autophagy* **10**, 70–79 (2014). 24262949 10.4161/auto.26534 PMC4389881

[48] Cho, H. Y., Loreti, E., Shih, M. C. & Perata, P. Energy and sugar signaling during hypoxia. *N. Phytol.* **229**, 57–63 (2021). 10.1111/nph.16326 31733144

[49] Papandreou, I., Cairns, R. A., Fontana, L., Lim, A. L. & Denko, N. C. HIF-1 mediates adaptation to hypoxia by actively downregulating mitochondrial oxygen consumption. *Cell Metab.* **3**, 187–197 (2006). 16517406 10.1016/j.cmet.2006.01.012

[50] Crabtree, H. G. Observations on the carbohydrate metabolism of tumours. *Biochem J.* **23**, 536–545 (1929). 16744238 10.1042/bj0230536 PMC1254097

[51] Rodriguez-Enriquez, S., Juarez, O., Rodriguez-Zavala, J. S. & Moreno-Sanchez, R. Multisite control of the Crabtree effect in ascites hepatoma cells. *Eur. J. Biochem* **268**, 2512–2519 (2001). 11298771 10.1046/j.1432-1327.2001.02140.x

[52] Marroquin, L. D., Hynes, J., Dykens, J. A., Jamieson, J. D. & Will, Y. Circumventing the Crabtree effect: replacing media glucose with galactose increases susceptibility of HepG2 cells to mitochondrial toxicants. *Toxicol. Sci.* **97**, 539–547 (2007). 17361016 10.1093/toxsci/kfm052

[53] Blackledge, N. P. et al. CpG islands recruit a histone H3 lysine 36 demethylase. *Mol. Cell* **38**, 179–190 (2010). 20417597 10.1016/j.molcel.2010.04.009 PMC3098377

[54] Huang, D. W., Sherman, B. T. & Lempicki, R. A. Systematic and integrative analysis of large gene lists using DAVID bioinformatics resources. *Nat. Protoc.* **4**, 44–57 (2009). 19131956 10.1038/nprot.2008.211

[55] Rath, S. et al. MitoCarta3.0: an updated mitochondrial proteome now with sub-organelle localization and pathway annotations. *Nucleic Acids Res* **49**, D1541–D1547 (2021). 33174596 10.1093/nar/gkaa1011 PMC7778944

[56] Kitamura, D., Roes, J., Kuhn, R. & Rajewsky, K. A B cell-deficient mouse by targeted disruption of the membrane exon of the immunoglobulin mu chain gene. *Nature* **350**, 423–426 (1991). 1901381 10.1038/350423a0

[57] Thai, T. H. et al. Regulation of the germinal center response by microRNA-155. *Science* **316**, 604–608 (2007). 17463289 10.1126/science.1141229

[58] Vigorito, E. et al. microRNA-155 regulates the generation of immunoglobulin class-switched plasma cells. *Immunity* **27**, 847–859 (2007). 18055230 10.1016/j.immuni.2007.10.009 PMC4135426

[59] Rodriguez, A. et al. Requirement of bic/microRNA-155 for normal immune function. *Science* **316**, 608–611 (2007). 17463290 10.1126/science.1139253 PMC2610435

[60] Hsin, J. P., Lu, Y., Loeb, G. B., Leslie, C. S. & Rudensky, A. Y. The effect of cellular context on miR-155-mediated gene regulation in four major immune cell types. *Nat. Immunol.* **19**, 1137–1145 (2018). 30224821 10.1038/s41590-018-0208-x PMC6158091

[61] Pasini, D. et al. JARID2 regulates binding of the Polycomb repressive complex 2 to target genes in ES cells. *Nature* **464**, 306–310 (2010). 20075857 10.1038/nature08788

[62] Schneider, C. et al. FBXO11 inactivation leads to abnormal germinal-center formation and lymphoproliferative disease. *Blood* **128**, 660–666 (2016). 27166359 10.1182/blood-2015-11-684357 PMC9709922

[63] Concordet, J. P. & Haeussler, M. CRISPOR: intuitive guide selection for CRISPR/Cas9 genome editing experiments and screens. *Nucleic Acids Res* **46**, W242–W245 (2018). 29762716 10.1093/nar/gky354 PMC6030908

[64] Paus, D. et al. Antigen recognition strength regulates the choice between extrafollicular plasma cell and germinal center B cell differentiation. *J. Exp. Med* **203**, 1081–1091 (2006). 16606676 10.1084/jem.20060087 PMC2118299

[65] Long, Z., Phillips, B., Radtke, D., Meyer-Hermann, M. & Bannard, O. Competition for refueling rather than cyclic reentry initiation evident in germinal centers. *Sci. Immunol.* **7**, eabm0775 (2022). 35275753 10.1126/sciimmunol.abm0775 PMC7614495

[66] Stirling, D. R. et al. CellProfiler 4: improvements in speed, utility and usability. *BMC Bioinforma.* **22**, 433 (2021). 10.1186/s12859-021-04344-9 PMC8431850 34507520

[67] Schindelin, J. et al. Fiji: an open-source platform for biological-image analysis. *Nat. Methods* **9**, 676–682 (2012). 22743772 10.1038/nmeth.2019 PMC3855844

[68] Legland, D., Arganda-Carreras, I. & Andrey, P. MorphoLibJ: integrated library and plugins for mathematical morphology with ImageJ. *Bioinformatics* **32**, 3532–3534 (2016). 27412086 10.1093/bioinformatics/btw413

[69] MacLean, B. et al. Skyline: an open source document editor for creating and analyzing targeted proteomics experiments. *Bioinformatics* **26**, 966–968 (2010). 20147306 10.1093/bioinformatics/btq054 PMC2844992

[70] Li, B. & Dewey, C. N. RSEM: accurate transcript quantification from RNA-Seq data with or without a reference genome. *BMC Bioinforma.* **12**, 323 (2011). 10.1186/1471-2105-12-323 PMC3163565 21816040

[71] Langmead, B. & Salzberg, S. L. Fast gapped-read alignment with Bowtie 2. *Nat. Methods* **9**, 357–359 (2012). 22388286 10.1038/nmeth.1923 PMC3322381

[72] Love, M. I., Huber, W. & Anders, S. Moderated estimation of fold change and dispersion for RNA-seq data with DESeq2. *Genome Biol.* **15**, 550 (2014). 25516281 10.1186/s13059-014-0550-8 PMC4302049

[73] R Core Team. R: A language and environment for statistical computing. R foundation for statistical computing 2021. https://www.R-project.org/ (2021).

[74] Subramanian, A. et al. Gene set enrichment analysis: a knowledge-based approach for interpreting genome-wide expression profiles. *Proc. Natl Acad. Sci. USA* **102**, 15545–15550 (2005). 16199517 10.1073/pnas.0506580102 PMC1239896

[75] Merico, D., Isserlin, R., Stueker, O., Emili, A. & Bader, G. D. Enrichment map: a network-based method for gene-set enrichment visualization and interpretation. *PLoS One* **5**, e13984 (2010). 21085593 10.1371/journal.pone.0013984 PMC2981572

[76] Chen, Y. & Wang, X. miRDB: an online database for prediction of functional microRNA targets. *Nucleic Acids Res* **48**, D127–D131 (2020). 31504780 10.1093/nar/gkz757 PMC6943051

[77] Vlachos, I. S. et al. DIANA-TarBase v7.0: indexing more than half a million experimentally supported miRNA:mRNA interactions. *Nucleic Acids Res* **43**, D153–D159 (2015). 25416803 10.1093/nar/gku1215 PMC4383989

[78] Agarwal, V., Bell, G. W., Nam, J. W. & Bartel, D. P. Predicting effective microRNA target sites in mammalian mRNAs. *Elife* **4**, e05005 (2015). 26267216 10.7554/eLife.05005 PMC4532895

[79] Satija, R., Farrell, J. A., Gennert, D., Schier, A. F. & Regev, A. Spatial reconstruction of single-cell gene expression data. *Nat. Biotechnol.* **33**, 495–502 (2015). 25867923 10.1038/nbt.3192 PMC4430369

[80] McGinnis, C. S., Murrow, L. M. & Gartner, Z. J. DoubletFinder: doublet detection in single-cell RNA sequencing data using artificial nearest neighbors. *Cell Syst.* **8**, 329–337.e324 (2019). 30954475 10.1016/j.cels.2019.03.003 PMC6853612

[81] Lun, A. T., McCarthy, D. J. & Marioni, J. C. A step-by-step workflow for low-level analysis of single-cell RNA-seq data with Bioconductor. *F1000Res* **5**, 2122 (2016). 27909575 10.12688/f1000research.9501.1 PMC5112579

[82] Li, H. & Durbin, R. Fast and accurate short read alignment with Burrows-Wheeler transform. *Bioinformatics* **25**, 1754–1760 (2009). 19451168 10.1093/bioinformatics/btp324 PMC2705234

[83] Zhang, Y. et al. Model-based analysis of ChIP-Seq (MACS). *Genome Biol.* **9**, R137 (2008). 18798982 10.1186/gb-2008-9-9-r137 PMC2592715

[84] Heinz, S. et al. Simple combinations of lineage-determining transcription factors prime cis-regulatory elements required for macrophage and B cell identities. *Mol. Cell* **38**, 576–589 (2010). 20513432 10.1016/j.molcel.2010.05.004 PMC2898526

[85] Ramirez, F. et al. deepTools2: a next generation web server for deep-sequencing data analysis. *Nucleic Acids Res* **44**, W160–W165 (2016). 27079975 10.1093/nar/gkw257 PMC4987876

[86] Karolchik, D. et al. The UCSC Table Browser data retrieval tool. *Nucleic Acids Res* **32**, D493–D496 (2004). 14681465 10.1093/nar/gkh103 PMC308837

