# Supplementary material for: Epi-microRNA mediated metabolic reprogramming counteracts hypoxia to preserve affinity maturation
Source: Nat Commun. 2024 Dec 3;15:10516. doi: 10.1038/s41467-024-54937-0 (PMC11615350; doi:10.1038/s41467-024-54937-0)
Supplement: Supplementary file 1 — Supplementary Information [file 41467_2024_54937_MOESM1_ESM.pdf]

Epi-microRNA mediated metabolic reprogramming counteracts hypoxia to preserve affinity maturation

Rinako Nakagawa[1*], Miriam Llorian[2], Sunita Varsani-Brown[3], Probir Chakravarty[2], Jeannie M. Camarillo[4], David Barry[5], Roger George[6], Neil P. Blackledge[7], Graham Duddy[3], Neil L. Kelleher[4], Robert J. Klose[7], Martin Turner[8] and Dinis P. Calado[1*]

1. Immunity and Cancer Laboratory, Francis Crick Institute, London, NW1 1AT, UK
2. Bioinformatics and Biostatistics Laboratory, Francis Crick Institute, London, NW1 1AT, UK
3. Genetic Modification Service Laboratory, Francis Crick Institute, London, NW1 1AT, UK
4. Department of Chemistry, Molecular Biosciences and the National Resource for Translational and Developmental Proteomics, Northwestern University, Evanston, IL, 60208, USA
5. Advanced Light Microscopy Laboratory, Francis Crick Institute, London, NW1 1AT, UK
6. Structural Biology Laboratory, Francis Crick Institute, London, NW1 1AT, UK
7. Department of Biochemistry, University of Oxford, Oxford, OX1 3QU, UK
8. Immunology Programme, The Babraham Institute, Cambridge, CB22 3AT, UK

* Correspondence: rinako.nakagawa@crick.ac.uk or dinis.calado@crick.ac.uk

Supplementary Materials

Supplementary figures

Supplementary Fig. 1

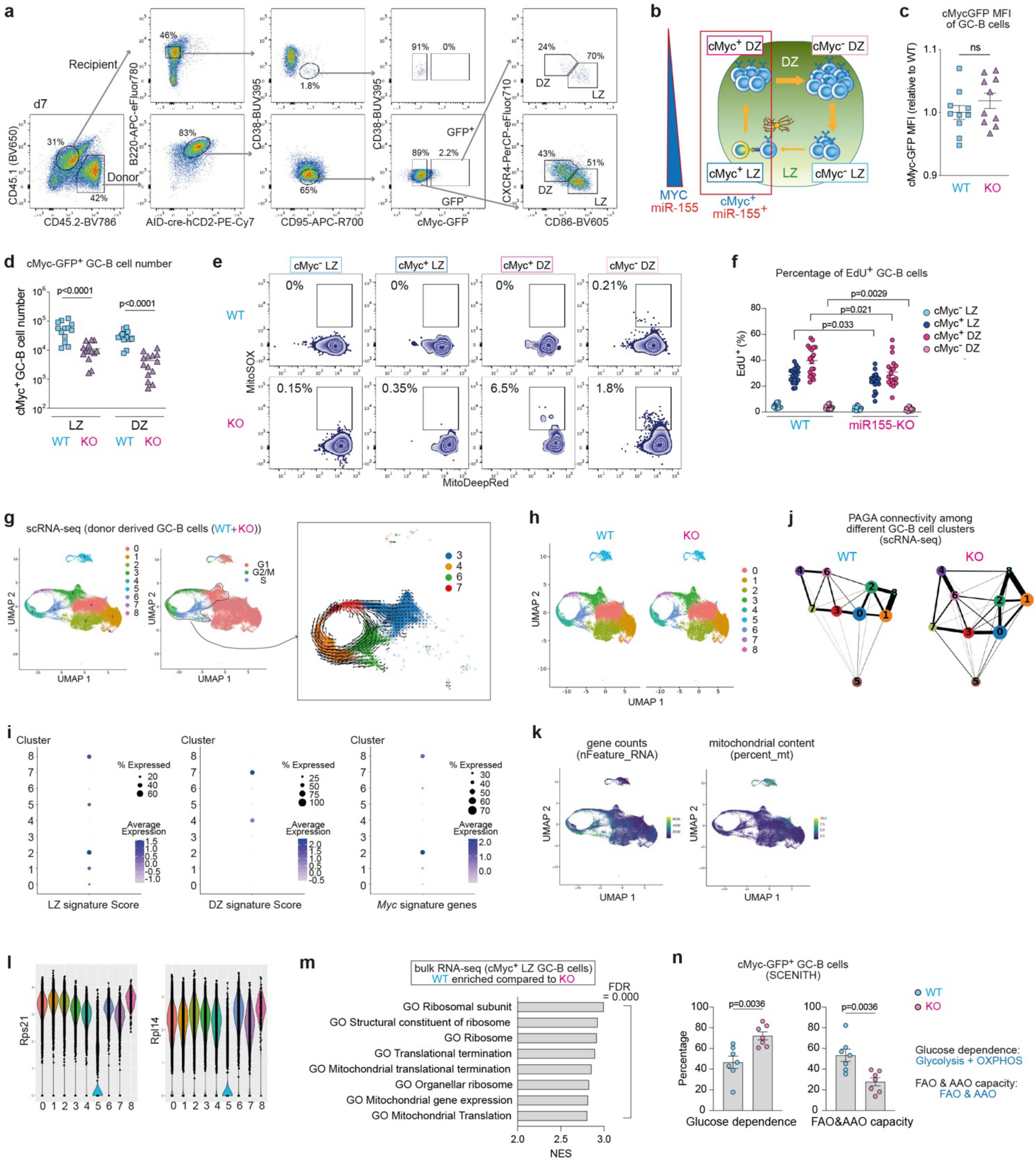

Supplementary Fig. 1. Gene expression patterns of miR-155-sufficient and miR-155-difficient GC-B cell clusters

a, Flow cytometry gating strategy for isolating splenic donor GC-B cells seven days after $HEL^{3\times}$-SRBC immunization. This gating strategy was applied for all SW_{HEL}-derived GC-B cells analyses in this study. **b**, Schematics illustrating the relationship between four GC-B cell subpopulations ($cMyc^-$ LZ, $cMyc^+$ LZ, $cMyc^+$ DZ and $cMyc^-$ DZ) and the expression patterns of cMyc and miR-155. **c**, MFI of cMyc-GFP in GC-B cells from WT and KO mice, eight days after $HEL^{3\times}$-SRBC immunization. Each dot represents one mouse. Paired student's t-test, two-tailed. Pooled from two experiments (WT n=11; KO n=11). **d**, Quantification of $cMyc^+$ LZ and DZ GC-B cell numbers in WT and KO mice, five days after $HEL^{3\times}$-SRBC immunization. Paired student's t-test, two-tailed. Pooled from four experiments (WT n=13; KO n=14). **e**, Representative flow cytometric plots of MitoTracker DeepRed vs. MitoSOX in GC-B cells from WT and KO mice, five days after $HEL^{3\times}$-SRBC immunization, from three independent experiments. **f**, Percentage of EdU^+ GC-B cells at seven days after $HEL^{3\times}$-SRBC immunization. Paired student's t-test, two-tailed. Pooled from two independent experiments (WT n=19; KO n=18). **g**, UMAP plots displaying merged scRNA-seq profiles from WT and KO GC-B cells, with nine clusters and predicted cell cycle phases (left). RNA velocity stream of clusters 3, 4, 6 and 7 overlaid on the UMAP plot shown on the left (right). **h**, UMAP plots showing scRNA-seq profiles from WT or KO GC-B cells, with nine identified clusters. **i**, Dot plots colored by the normalized expression of signature genes associated with LZ, DZ and Myc signature genes. **j**, Ball-and-stick representation of PAGA connectivity among scRNA-seq GC-B cell clusters from WT or KO GC-B cells. **k**, UMAP plots colored by the number of genes detected in each cell or the percentage of mitochondrial genes. **l**, Violin plots illustrating the expression levels of Rps21 or Rpl14 in the GC-B cell clusters. **m**, Top eight GSEA gene signatures in WT vs. KO comparison using RNA-seq dataset of $cMyc^+$ LZ GC-B cells. WT-enriched gene signatures are shown. FDR; false discovery rate, NEM; normalized enrichment score. **n**, Quantification of glucose dependence and FAO or AAO capacity of GC-B cells from WT and KO mice based on puromycin incorporation for 15 minutes, treated with metabolic inhibitors (oligomycin or 2-deoxy-D-glucose). GC-B cells are derived from five days after $HEL^{3\times}$-SRBC immunization. Paired student's t-test, two-tailed. Pooled from three independent experiments (WT n=7; KO n=7). Unless otherwise stated, mean ± SEM is indicated. n.s., not significant.

Supplementary Fig. 2

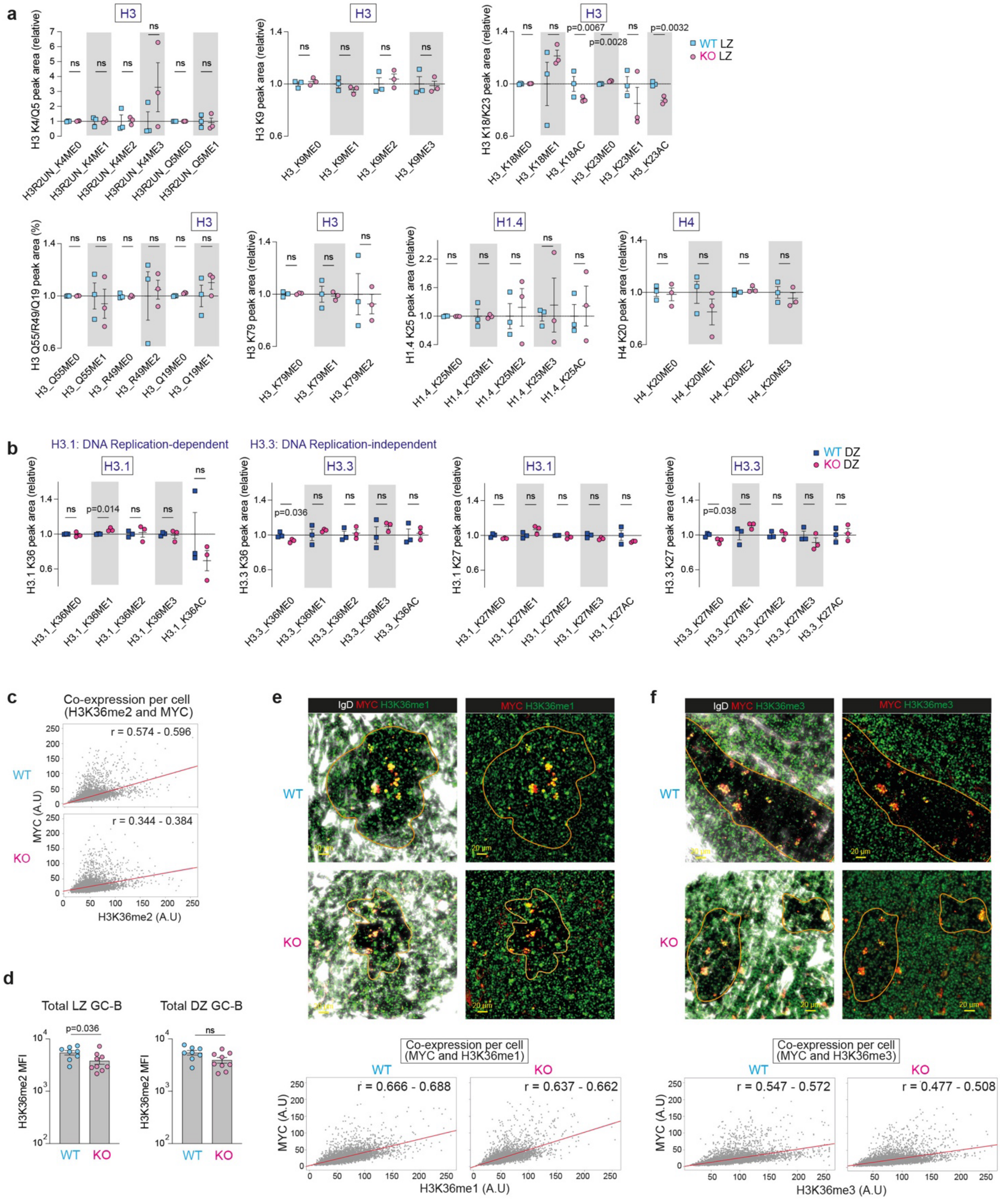

Supplementary Fig. 2. Histone PTM states in *Mir155*-sufficient and *Mir155*-deficient GC-B cells

a, The relative abundance of histone PTMs in LZ GC-B cells from WT and KO mice, analyzed in triplicate. Donor-derived GC-B cells were sorted five days after adoptive transfer for the analysis. Paired student's t-test, two-tailed. **b**, The relative abundance of histone PTMs, including histone H3.1 K36, H3.3 K36, H3.1 K27 and H3.3 K27, in DZ GC-B cells from WT and KO mice. Donor-derived GC-B cells were sorted five days after adoptive transfer for the analysis. Triplicate samples were used for both WT and KO backgrounds. Paired student's t-test, two-tailed. **c,** Scatter plots show the mean intensity (MI) of MYC and H3K36me2 per segmented cell within GCs of spleen sections obtained from immunized WT and KO C57BL/6 mice. Cell segmentation within GCs was performed using DAPI staining on the sections. The Pearson correlation coefficient indicates the co-expression relationship between MYC and H3K36me2. The corresponding coefficient value (r) and a 95% confidence interval is displayed. A.U., arbitrary unit. **d,** MFI of H3K36me2 in LZ GC-B cells (left) and DZ GC-B cells (right). Paired student's t-test, two-tailed. Pooled from three independent experiments (WT n=8; KO n=9). **e, f**, Immunofluorescence staining of splenic sections from WT mice 7 days after SRBC immunization, showing IgD (white), MYC (red) and H3K36me1 (**e**) or H3K36me3 (**f**) (green). Scale bar, 20 μm. Scatter plots illustrating the expression of MYC and H3K36me1(**e**) or H3K36me3 (**f**) in segmented GC cells within spleen sections from immunized WT and KO mice. Pearson correlation coefficient (r) indicates the co-expression between MYC and H3K36me1 (**e**) or H3K36me3 (**f**). The corresponding coefficient value (r) and a 95% confidence interval is displayed. Representative section images from two experiments (WT n=4; KO n=4). Unless otherwise stated, mean ± SEM is indicated. n.s., not significant.

Supplementary Fig. 3

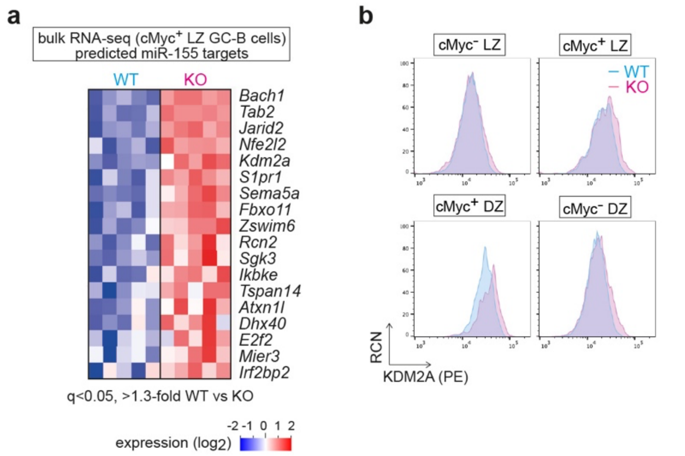

Supplementary Fig. 3. The histone demethylase *Kdm2a* is a target of miR-155

a, Heat map showing the transcript levels of the miR-155 targets. The miR-155 targets displaying a significant (FDR <0.05) and at least 1.3-fold increase in KO cMyc$^+$ LZ GC-B cells compared to WT are shown. The bulk RNA-seq dataset of WT and KO cMyc$^+$ LZ GC-B cells was used. **b**, Representative flow cytometric histograms showing KDM2A vs. relative cell number (RCN).

Supplementary Fig. 4

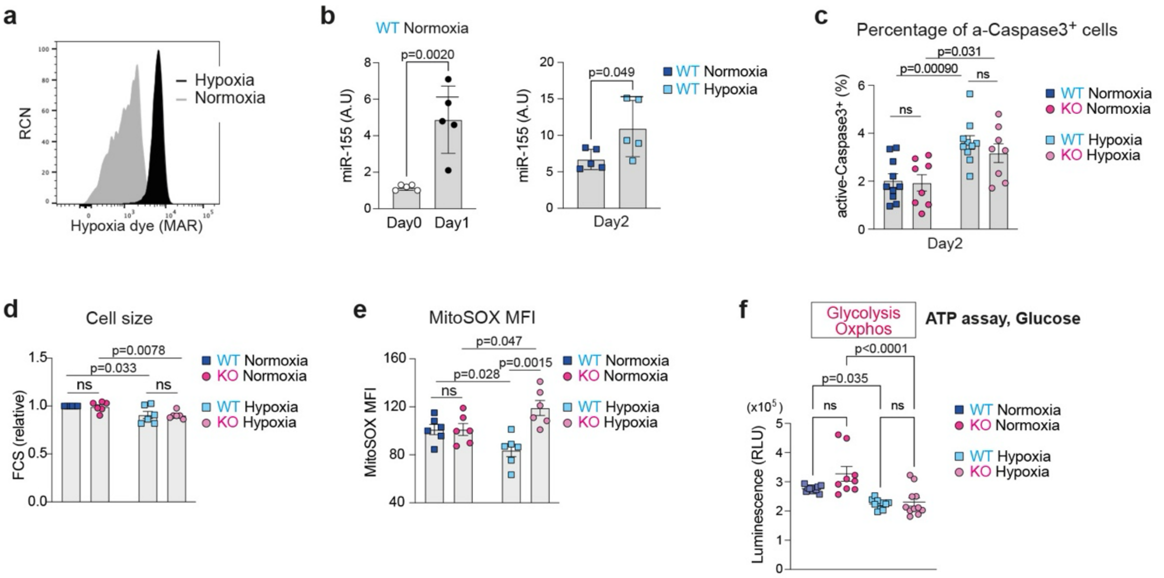

Supplementary Fig. 4. Induction of miR-155 by positive selection signals in normoxia and hypoxia

a, Representative flow cytometric histograms of hypoxia dye, MAR vs. RCN. Splenic B cells either cultured under either normoxic (21% O_2) or hypoxic (1% O_2) conditions are shown. **b**, MiR-155 expression levels in B cells cultured under normoxia and hypoxia. Paired student's t-test, two-tailed. Pooled data from two experiments (WT n=5; KO n=5). **c**, Percentage of active-Caspase 3^+ B cells from WT and KO mice cultured under normoxia or hypoxia. Paired student's t-test, two-tailed. Pooled from three independent experiments (WT n=10; KO n=8). **d**, Relative FCS values in B cells from WT and KO mice cultured under normoxia or hypoxia. Paired student's t-test, two-tailed. Pooled from three independent experiments (WT n=6; KO n=6). **e**, MFI of MitoSOX MFI in B cells from WT and KO mice cultured under normoxia or hypoxia. Paired student's t-test, two-tailed. Pooled from three independent experiments (WT n=6; KO n=6). **f**, Relative luminescence units (RLU) of B cells cultured in glucose containing media for two hours in hypoxia. One-way ANOVA. Pooled from three experiments (WT n=3; KO n=3). B cells in all experiments for these figures were stimulated with anti-IgM, anti-CD40 and IL-4. Unless otherwise stated, mean ± SEM is indicated. Each dot represents one mouse sample. n.s., not significant.

Supplementary Fig. 5

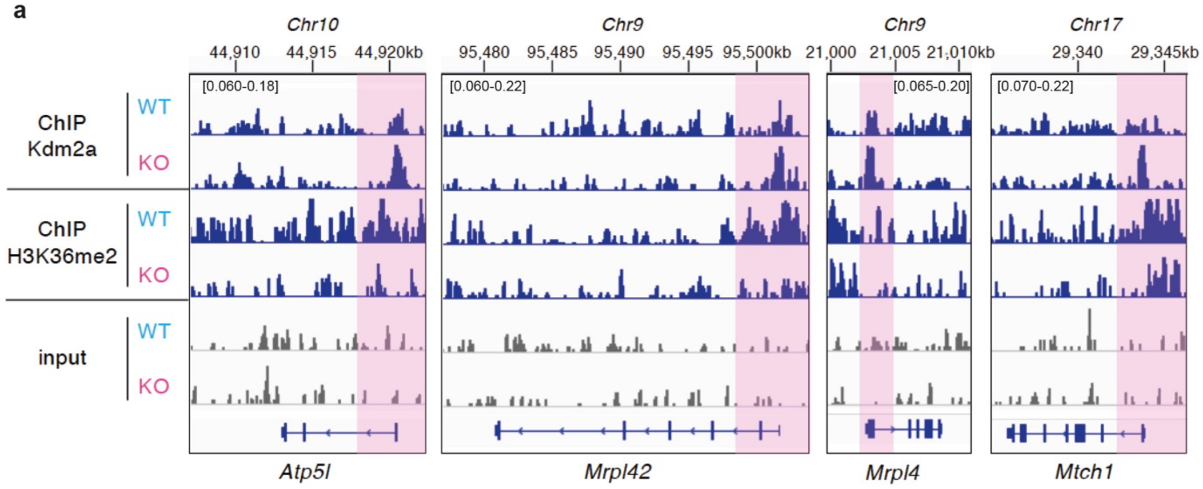

Supplementary Fig. 5. The genes with KO-enriched KDM2A peaks showed a decrease in H3K36me2 levels

a, IGV snapshots showing KDM2A and H3K36me2 ChIP-seq in hypoxic cultured B cells from WT and KO mice for representative nuclear mitochondrial genes with “KO-enriched KDM2A” peaks. The interval scales for the x-axis are indicated at the top of each gene track.

Supplementary Fig. 6

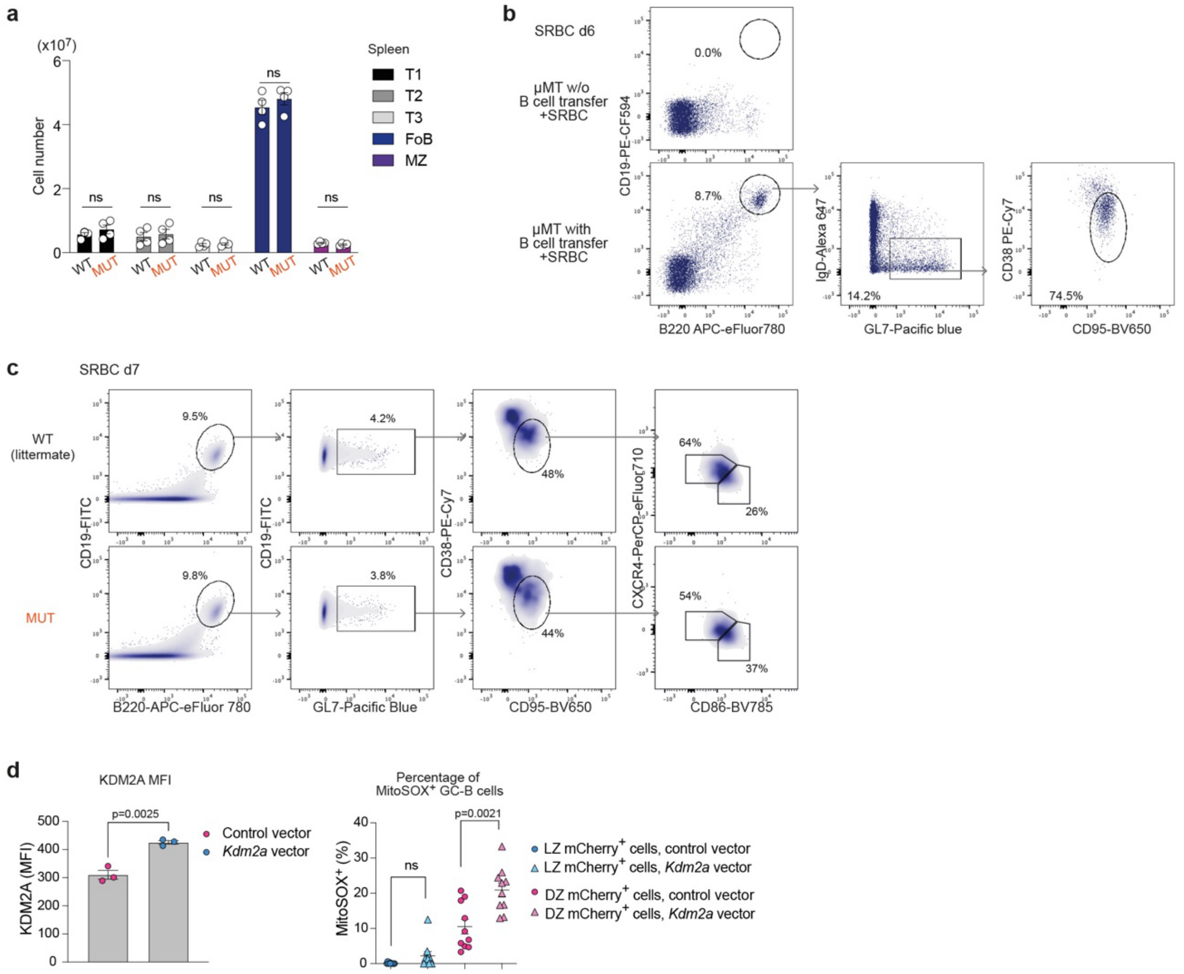

Supplementary Fig. 6. Increased KDM2A expression exacerbates mitochondrial ROS in the absence of miR-155

a, Total B lineage cell numbers in the spleen of WT and MUT mice. T1: $CD19^{+}$ $B220^{+}$ $AA4.1^{+}$ $CD23^{-}$ IgM^{+}; T2: $CD19^{+}$ $B220^{+}$ $AA4.1^{+}$ $CD23^{+}$ IgM^{+}; T3: $CD19^{+}$ $B220^{+}$ $AA4.1^{+}$ $CD23^{+}$ IgM^{low}; Follicular (Fo) B: $CD19^{+}$ $B220^{+}$ $AA4.1^{-}$ $CD23^{+}$ IgM^{low}; Marginal zone (MZ) B: $CD19^{+}$ $B220^{+}$ $AA4.1^{-}$ $CD23^{low}$ IgM^{+}. Paired student's t-test, two-tailed. Pooled from two experiments (WT n=4; MUT n=4). **b**, Representative flow cytometric plots showing the analysis of µMT mice transferred with or without donor B cells from a WT mouse six days after SRBC immunization. Recipient µMT mice without donor B cells failed to generate GC-B cells. **c**, Representative flow cytometric plots analyzing µMT mice transferred with WT or MUT B cells seven days after SRBC immunization. This gating strategy was applied for **Fig.6 b-e**. **d**, Activated SW_{HEL} B cells were transduced with *Kdm2a* or control (empty) lentivirus. MFI of KDM2A was analyzed in activated B cells cultured for two days following transduction. Representative of two independent experiments. Either control- or *Kdm2a*-transduced HEL-activated donor SW_{HEL} B cells were

transferred into congenic mice. GC-B cells derived from transduced donor cells were stained for MitoSOX four days after $HEL^{3\times}$-SRBC immunization (right). Paired student's t-test, two-tailed. Pooled from two experiments (Control n=10; *Kdm2a* n=10). Unless otherwise stated, mean ± SEM is indicated. n.s., not significant.

Supplementary Data

Supplementary Data 1. Identified cluster marker genes in scRNA-seq from total GC-B cells of WT and KO mice

The two-sided, adjusted p-value was calculated using Bonferroni correction based on the total number of genes in the dataset. P values less than 0.05 are considered as significant.

Supplementary Data 2. Differentially expressed genes in bulk RNA-seq of WT and KO cMyc^{+} LZ GC-B cells

The two-sided, adjusted p-value was calculated using Bonferroni correction based on the total number of genes in the dataset. P values less than 0.05 are considered as significant.

Supplementary Data 3. Gene lists within the gene sets enriched in KO cMyc^{+} LZ GC-B cells compared to WT cMyc^{+} LZ GC-B cells

Supplementary Data 4. H3K36me levels in the TSS regions of CpG genes: KO vs. WT

Supplementary Data 5. Genes with "WT-enriched" and "KO-enriched" KDM2A peaks in activated B cells under hypoxia
