# Supplementary material for: Epi-microRNA mediated metabolic reprogramming counteracts hypoxia to preserve affinity maturation
Source: Nat Commun. 2024 Dec 3;15:10516. doi: 10.1038/s41467-024-54937-0 (PMC11615350; doi:10.1038/s41467-024-54937-0)
Supplement: Supplementary file 2 — Description of Additional Supplementary Files [file 41467_2024_54937_MOESM2_ESM.pdf]

Description of Additional Supplementary Files

Supplementary Data 1. Identified cluster marker genes in scRNA-seq from total GC-B cells of WT and KO mice
The two-sided, adjusted p-value was calculated using Bonferroni correction based on the total number of genes in the dataset. P values less than 0.05 are considered as significant.

Supplementary Data 2. Differentially expressed genes in bulk RNA-seq of WT and KO cMyc$^+$ LZ GC-B cells
The two-sided, adjusted p-value was calculated using Bonferroni correction based on the total number of genes in the dataset. P values less than 0.05 are considered as significant.

Supplementary Data 3. Gene lists within the gene sets enriched in KO cMyc$^+$ LZ GC-B cells compared to WT cMyc$^+$ LZ GC-B cells

Supplementary Data 4. H3K36me levels in the TSS regions of CpG genes: KO vs. WT

Supplementary Data 5. Genes with “WT-enriched” and “KO-enriched” KDM2A peaks in activated B cells under hypoxia
