# Supplementary material for: Epi-microRNA mediated metabolic reprogramming counteracts hypoxia to preserve affinity maturation
Source: Nat Commun. 2024 Dec 3;15:10516. doi: 10.1038/s41467-024-54937-0 (PMC11615350; doi:10.1038/s41467-024-54937-0)
Supplement: Supplementary file 8 — Reporting Summary [file 41467_2024_54937_MOESM8_ESM.pdf]

nature portfolio

Corresponding author(s): Rinako Nakagawa, Dinis Calado

Last updated by author(s): 29/October/2024

Reporting Summary

Nature Portfolio wishes to improve the reproducibility of the work that we publish. This form provides structure for consistency and transparency in reporting. For further information on Nature Portfolio policies, see our Editorial Policies and the Editorial Policy Checklist.

Statistics

For all statistical analyses, confirm that the following items are present in the figure legend, table legend, main text, or Methods section.

n/a	Confirmed	
☐	☒	The exact sample size (*n*) for each experimental group/condition, given as a discrete number and unit of measurement
☐	☒	A statement on whether measurements were taken from distinct samples or whether the same sample was measured repeatedly
☐	☒	The statistical test(s) used AND whether they are one- or two-sided<br>*Only common tests should be described solely by name; describe more complex techniques in the Methods section.*
☒	☐	A description of all covariates tested
☐	☒	A description of any assumptions or corrections, such as tests of normality and adjustment for multiple comparisons
☐	☒	A full description of the statistical parameters including central tendency (e.g. means) or other basic estimates (e.g. regression coefficient) AND variation (e.g. standard deviation) or associated estimates of uncertainty (e.g. confidence intervals)
☐	☒	For null hypothesis testing, the test statistic (e.g. *F*, *t*, *r*) with confidence intervals, effect sizes, degrees of freedom and *P* value noted<br>*Give P values as exact values whenever suitable.*
☒	☐	For Bayesian analysis, information on the choice of priors and Markov chain Monte Carlo settings
☒	☐	For hierarchical and complex designs, identification of the appropriate level for tests and full reporting of outcomes
☐	☒	Estimates of effect sizes (e.g. Cohen's *d*, Pearson's *r*), indicating how they were calculated

Our web collection on statistics for biologists contains articles on many of the points above.

Software and code

Policy information about availability of computer code

Data collection	Spark multimode microplate reader (TECAN); LSRFortessa, LSRFortessa X20 flow cytometers (BD Biosciences); FACSAria and Fusion cell sorters (BD Biosciences); LSM880 inverted confocal microscope (Zeiss), Thermo Scientific TSQ Quantiva Mass Sectrometer (Thermo Fisher Scientific); NovaSeq 6000 (Illumina).
Data analysis	GraphPad Prism (v.9.4.1) (GraphPad); FlowJo (v.10.8.1) (BD); Lasergene 17.4 (DNASTAR); GSEA (v.2.1.0)(Broad Institute); ImageJ FIJI (v.2.9.0) (https://imagej.net/software/fiji/); Zen 3 Microscopy software (Zeiss); CellProfiler (v.4.2.5) (Broad Institute); Skyline (v.21.2) (MacCoss Lab Software); R (v.3.6.0) (https://www.r-project.org/).

For manuscripts utilizing custom algorithms or software that are central to the research but not yet described in published literature, software must be made available to editors and reviewers. We strongly encourage code deposition in a community repository (e.g. GitHub). See the Nature Portfolio guidelines for submitting code & software for further information.

Data

Policy information about availability of data

All manuscripts must include a data availability statement. This statement should provide the following information, where applicable:

- Accession codes, unique identifiers, or web links for publicly available datasets
- A description of any restrictions on data availability
- For clinical datasets or third party data, please ensure that the statement adheres to our policy

The scRNA-seq, bulk RNA-seq and ChIP-seq data have been deposited in the NCBU Gene Expression Omnibus (GEO) database under accession number GSE230528, GSE231649, GSE230527, respectively. Source data are provided with this paper.

Research involving human participants, their data, or biological material

Policy information about studies with human participants or human data. See also policy information about sex, gender (identity/presentation), and sexual orientation and race, ethnicity and racism.

Reporting on sex and gender	N/A
Reporting on race, ethnicity, or other socially relevant groupings	N/A
Population characteristics	N/A
Recruitment	N/A
Ethics oversight	N/A

Note that full information on the approval of the study protocol must also be provided in the manuscript.

Field-specific reporting

Please select the one below that is the best fit for your research. If you are not sure, read the appropriate sections before making your selection.

☒ Life sciences ☐ Behavioural & social sciences ☐ Ecological, evolutionary & environmental sciences

For a reference copy of the document with all sections, see nature.com/documents/nr-reporting-summary-flat.pdf

Life sciences study design

All studies must disclose on these points even when the disclosure is negative.

Sample size	Sample sizes were determined based on the experimental design, previous similar experiments conducted in the laboratory, and relevant literature to ensure statistical significance. Details on sample size and the number of independent experiments are provided in the figure legends.
Data exclusions	No data were excluded from the analyses unless indicated in methods or figure legends.
Replication	Experiments were conducted with at least two independent times. The sample size and number of independent experiments are indicated in figure legends.
Randomization	Randomization was not applicable to the experiments comparing cell populations within animals of the same genotype. Age matched mice were used for all experiments.
Blinding	No blinding was performed as mice were grouped by genotype.

Reporting for specific materials, systems and methods

We require information from authors about some types of materials, experimental systems and methods used in many studies. Here, indicate whether each material, system or method listed is relevant to your study. If you are not sure if a list item applies to your research, read the appropriate section before selecting a response.

Materials & experimental systems

n/a	Involved in the study
	☒ Antibodies
☒	Eukaryotic cell lines
☒	Palaeontology and archaeology
	☒ Animals and other organisms
☒	Clinical data
☒	Dual use research of concern
☒	Plants

Methods

n/a	Involved in the study
	☒ ChIP-seq
	☒ Flow cytometry
☒	MRI-based neuroimaging

Antibodies

Antibodies used	CD43, biotin (S7), BD Biosciences, 553269; CD4, biotin (GK1.5), BioLegend, 100404; Ter119, biotin (TER-119), BioLegend, 116204; CD45.1, biotin (A20), ThermoFisher Sciences, 13-0453-85; CD21/35, BV786 (7G6), BD Biosciences, 740894; CD23, FITC (B3B4), BD Biosciences, 553138; CD93, PE (AA4.1), ThermoFisher Sciences, 12-5892-82; CXCR4, PerCP-eFluor710 (2B11), ThermoFisher Sciences, 46-9991-82; CXCR4, APC (2B11), BD Biosciences, 558644; CD86, FITC (GL1), BD Biosciences, 553691; CD86, PECy7 (GL1), BioLegend, 105014; CD86, APCCy7 (GL1), BioLegend, 105030; CD86, BV605 (GL1), BioLegend, 105037; CD86, BV785 (GL1), BioLegend, 105043; CD95, PECy7 (Jo2), BD Biosciences, 557653; CD95, APC-R700 (Jo2), BD Biosciences, 565130; CD95, BV786 (Jo2), BD Biosciences, 740906; CD38, PerCP-eFluor710 (90), ThermoFisher Sciences, 46-0381-82; CD38, PECy7 (90), BioLegend, 102718; CD38, BV650 (90), BD Biosciences, 740489; CD38, BUV395 (90), BD Biosciences, 740245; GL7, Pacific blue (GL7), BioLegend, 144614; GL7, Alexa Fluor 488 (GL7), ThermoFisher Sciences, 53-5902-82; human CD2, PECy7 (TS1/8), BioLegend, 309214; human CD2, APC (TS1/8), BioLegend, 309224; human CD2, Alexa700 (TS1/8), BioLegend, 309228; CD45.2, BV605 (104), BD Biosciences, 563051; CD45.2, BV786 (104), BD Biosciences, 563686; CD45.2, BUV395 (104), BD Biosciences, 564616; CD138, PE, (281-2), BioLegend, 142504; CD138, APC, (281-2), BioLegend, 142506; CD138, APCCy7, (281-2), BioLegend, 142530; CD138, BV605, (281-2), BioLegend, 142531; CD138, BV786, (281-2), BD Biosciences, 740880; B220, FITC (RA3-6B2), ThermoFisher Sciences, 11-0452-82; B220, APCCy7 (RA3-6B2), ThermoFisher Sciences, 47-0452-82; B220, BV421 (RA3-6B2), BioLegend, 103240; B220, BV650 (RA3-6B2), BioLegend, 103241; B220, BUV787 (RA3-6B2), BD Biosciences, 612838; CD19, FITC (6D5), BioLegend, 115506; CD19, BV605 (6D5), BioLegend, 115540; CD19, BV650 (6D5), BioLegend, 115541; CD19, PE-CF594 (1D3), BD Biosciences, 562291; IgM, BV605 (II/41), BD Biosciences, 743325; IgD, Alexa Fluor 647 (11-26c.2a), BioLegend, 405708; IgG1, FITC (A85-1), BD Biosciences, 553443; IgG1, CF-594 (A85-1), BD Biosciences, 562559; Igk light chain, BV421 (187.1), BD Biosciences, 562888; rabbit IgG, Alexa Fluor 647 (Poly4064), BioLegend, 406414; rabbit IgG, PE (Poly4064), BioLegend, 406421; rabbit IgG, Alexa Fluor 488 (Poly4064), BioLegend, 406416; CD40, purified (HM-40), BioLegend, 102902; active caspase 3, PE (C92-605), BD Biosciences, 550821; KDM2A, purified (EPR18602), Abcam, ab191387; cMYC, Alexa Fluor 555 (9E10), ThermoFisher Sciences, MA1-980-A555; Histone H3K36me2, purified (EPR16994(2)), Abcam, ab176921; mouse IgM F(ab')2 fragment, Jackson ImmunoResearch, 115-006-020; Streptavidin, PerCP-Cy5.5, BioLegend, 405214; Streptavidin, PE, ThermoFisher Sciences, 12-4317-87; Streptavidin, BV650, BioLegend, 405231; Streptavidin, BUV737, BD Biosciences, 612775.
Validation	All antibodies, except for the KDM2A antibody used for ChIP-seq, are commonly used clones that are commercially available and validated both by the manufacturer, cited in the scientific literature, or used in previous experiments. Validation materials for each antibody can be accessed on the respective manufacturer's webpage. The KDM2A antibody used for ChIP-seq was validated and used for ChIP-seq (PMID: 20417597).

Animals and other research organisms

Policy information about studies involving animals; ARRIVE guidelines recommended for reporting animal research, and Sex and Gender in Research

Laboratory animals	B6.Cg-Tyr c-Brd Mir155 tm1.1Brd /H mouse (PMID: 17463290); Ightm1Rbr (SWHEL heavy chain) mouse and Tg(IgkHyHEL10)1Rbr (SWHEL light chain) mouse (PMID: 12668643); Tg(Aicda-cre)9Mbu (Aicda-cre-hCD2) mouse (PMID: 18538592); Myctm1Slek (Mycgfp/gfp) mouse (PMID: 12668643); B6.129S2-Ighmtm1Cgn/J mouse (Strain #2288, The Jackson Laboratories); B6.SJL.CD45.1.J mouse (Strain #494, CHarles River Laboratories) were all on a C57BL/6J (Strain #664, The Jackson Laboratories). 6 - 25 week old mice were used in the experiments.
Wild animals	This study did not involve wild animals.
Reporting on sex	The experiments in this study contain mice of both sexes, so the findings are not limited to one sex. However, the GC-B cells used for bulk RNA-seq experiments were predominantly from male mice in KO background and female mice in WT background, as described in the Methods section.
Field-collected samples	This study did not involve samples collected from the field.
Ethics oversight	All animal procedures reported in this study were performed by staff at the Francis Crick Institute and were approved by the Francis Crick Institute Biological Research Facility in accordance with guidelines set by UK Home Office and the Francis Crick Institute Ethical Review Panel.

Note that full information on the approval of the study protocol must also be provided in the manuscript.

Plants

Seed stocks

N/A

Novel plant genotypes

N/A

Authentication

N/A

ChIP-seq

Data deposition

☒ Confirm that both raw and final processed data have been deposited in a public database such as GEO.

☒ Confirm that you have deposited or provided access to graph files (e.g. BED files) for the called peaks.

Data access links
May remain private before publication.

https://www.ncbi.nlm.nih.gov/geo/query/acc.cgi?&acc=GSE230527

Files in database submission

GSM7226214_WT_H3K36me2_R1.bigWig
GSM7226214_WT_H3K36me2_R1_peaks.broadPeak.gz
GSM7226215_WT_H3K36me2_R2.bigWig
GSM7226215_WT_H3K36me2_R2_peaks.broadPeak.gz
GSM7226216_WT_H3K36me2_R3.bigWig
GSM7226216_WT_H3K36me2_R3_peaks.broadPeak.gz
GSM7226217_KO_H3K36me2_R1.bigWig
GSM7226217_KO_H3K36me2_R1_peaks.broadPeak.gz
GSM7226218_KO_H3K36me2_R2.bigWig
GSM7226218_KO_H3K36me2_R2_peaks.broadPeak.gz
GSM7226219_KO_H3K36me2_R3.bigWig
GSM7226219_KO_H3K36me2_R3_peaks.broadPeak.gz
GSM7226220_WT_Kdm2a_R1.bigWig
GSM7226220_WT_Kdm2a_R1_peaks.broadPeak.gz
GSM7226221_WT_Kdm2a_R2.bigWig
GSM7226221_WT_Kdm2a_R2_peaks.broadPeak.gz
GSM7226222_WT_Kdm2a_R3.bigWig
GSM7226222_WT_Kdm2a_R3_peaks.broadPeak.gz
GSM7226223_KO_Kdm2a_R1.bigWig
GSM7226223_KO_Kdm2a_R1_peaks.broadPeak.gz
GSM7226224_KO_Kdm2a_R2.bigWig
GSM7226224_KO_Kdm2a_R2_peaks.broadPeak.gz
GSM7226225_KO_Kdm2a_R3.bigWig
GSM7226225_KO_Kdm2a_R3_peaks.broadPeak.gz
GSM7226226_WT_input_R1.bigWig
GSM7226227_WT_input_R2.bigWig
GSM7226228_WT_input_R3.bigWig
GSM7226229_KO_input_R1.bigWig
GSM7226230_KO_input_R2.bigWig
GSM7226231_KO_input_R3.bigWig

Genome browser session
(e.g. UCSC)

N/A

Methodology

Replicates

ChIP-seq experiments were profiled with triplicate samples.

Sequencing depth

ChIP-seq were 50bp paired-end.

sample name: Total peak count
WT_H3K36me2_R1: 85386
WT_H3K36me2_R2: 89767
WT_H3K36me2_R3: 94135
WT_Kdm2a_R1: 29104
WT_Kdm2a_R2: 23371

WT_Kdm2a_R3: 26830
KO_Kdm2a_R1: 15666
KO_Kdm2a_R2: 15386
KO_Kdm2a_R3: 21704
KO_H3K36me2_R1: 106719
KO_H3K36me2_R2: 37446
KO_H3K36me2_R3: 45179

Antibodies

Histone H3K36me2 antibody (EPR16994(2)), Abcam; KDM2A antibody (PMID: 20417597).

Peak calling parameters

macs2 callpeak -t IP.bam -c control.bam --broad --broad-cutoff 0.1 -f BAM -g 1.87e9 -B --SPMR --min_reps_consensus 2 --keep-dup all

Data quality

ChIP-seq data quality was confirmed through various assessments. Fastqc was executed on all samples to ensure quality data. The number of peaks was with over 3-fold enrichment and a cutoff of FDR <= 0.05 are as follows:
samples peaks
WT_H3K36me2_R1 :4214
WT_H3K36me2_R2 :1421
WT_H3K36me2_R3 :1612
KO_H3K36me2_R1:14414
KO_H3K36me2_R2 :1000
KO_H3K36me2_R3 :3191
WT_Kdm2a_R1 :4655
WT_Kdm2a_R2 :8584
WT_Kdm2a_R3 :5814
KO_Kdm2a_R1 :4290
KO_Kdm2a_R2 :3502
KO_Kdm2a_R3 :4771

Software

MACS2 (v.2.2.7) and HOMER (v.4.11)

Flow Cytometry

Plots

Confirm that:

☒ The axis labels state the marker and fluorochrome used (e.g. CD4-FITC).

☒ The axis scales are clearly visible. Include numbers along axes only for bottom left plot of group (a 'group' is an analysis of identical markers).

☐ All plots are contour plots with outliers or pseudocolor plots.

☒ A numerical value for number of cells or percentage (with statistics) is provided.

Methodology

Sample preparation

For live cell staining, harvested cells were incubated with with antibodies for surface antigens for 15-30 min on ice and then washed twice with FACS buffer. For CD45.1 staining, cells were subsequently stained with fluorescent streptavidin for 15-20 min on ice and washed twice with FACS buffer.
For intracellular staining, after the surface antigen staining, cells were washed with FACS buffer and fixed and permeabilized using a Fixation/Permeabilization Solution Kit (BD Biosciences) for active-caspase 3 staining or a True-Nuclear Transcription Factor Buffer Set (BioLegend) for KDM2A and H3K36me2 staining. The samples were incubated for 30 min at 4°C. Cells were then washed twice with Perm/Wash buffer (BD Biosciences) for active-caspase 3 staining or with True-Nuclear perm buffer (BioLegend) for KDM2A/H3K36me2 staining. Next, the samples were incubated with antibodies against intracellular antigens diluted in Perm/Wash buffer or True-Nuclear perm buffer for 30 min - overnight at 4°C. Beofre flow cytometric acquisition, the cells were washed twice with Perm/Wash buffer or True-Nuclear perm buffer.

Instrument

LSRFortessa, LSRFortessa X20 flow cytometers (BD Biosciences); FACSAria and Fusion cell sorters (BD Biosciences)

Software

FlowJo (v.10.8.1) (BD)

Cell population abundance

In all the experiments, an adequate number of cells were acquired from on sample (~ 2,500,000). cMYC+ GC-B cells typically constitute less than 10 % of total GC-B cells. Therefore, in the SWHEL adoptive transfer system, the total number of cMYC+ GC-B cells ranged from 100 to 500 cells after acquiring all cells following MACS purification.

Gating strategy

Also see Supplementary Fig. 1a and Supplementary Fig. 6b and c. Debris were excluding using FSC-A/SSC-A gates and aggregates were excluded using FSC-W/FSC-A and SSC-W/SSC-A gates. Dead cells were excluded by gating on cells lacking staining with DAPI or Zombie-NIR.
In the SWHEL adoptive transfer system, donor-derived GC-B cells were typically gated as CD45.2+ CD45.1- CD19+ B220+ CD38lo CD95+ hCD2+. DZ GC-B cells were gated as CXCR4hi CD86lo, while LZ GC-B cells were gated as CXCR4lo CD86hi. MYC positivity was determined by GFP expression in the cMyc-GFP reporter,with recipient-derived GC-B cells serving as the negative indicator for GFP.

☒ Tick this box to confirm that a figure exemplifying the gating strategy is provided in the Supplementary Information.
